# Elucidation of Galactomannan Biosynthesis Pathway Genes through Transcriptome Sequencing of Seeds Collected at Different Developmental Stages of Commercially Important Indian Varieties of Cluster Bean (*Cyamopsis tetragonoloba* L.)

**DOI:** 10.1038/s41598-019-48072-w

**Published:** 2019-08-08

**Authors:** Ashok Chaudhury, Tanvi Kaila, Kishor Gaikwad

**Affiliations:** 1 Plant Molecular Biology Laboratory, Department of Bio and Nano Technology, Bio and Nano Technology Centre, Guru Jambheshwar University of Science and Technology, Hisar, 125 001 Haryana India; 2 ICAR-National Research Centre on Plant Biotechnology, LBS Centre, Pusa Campus, New Delhi, 110 012 India

**Keywords:** Gene expression profiling, Transcriptomics, Gene ontology

## Abstract

*Cyamopsis tetragonoloba* (L) endosperm predominantly contains guar gum a polysaccharide, which has tremendous industrial applications in food, textile, paper, oil drilling and water treatment. In order to understand the genes controlling galactomannan biosynthesis, mRNA was isolated from seeds collected at different developmental stages; young pods, mature pods and young leaf from two guar varieties, HG365 and HG870 and subjected to Illumina sequencing. *De novo* assembly of fourteen individual read files from two varieties of guar representing seven developmental stages gave a total of 1,13,607 contigs with an N50 of 1,244 bases. Annotation of assemblies with GO mapping revealed three levels of distribution, namely, Biological Processes, Molecular Functions and Cellular Components. GO studies identified major genes involved in galactomannan biosynthesis: Cellulose synthase D1 (CS D1) and GAUT-like gene families. Among the polysaccharide biosynthetic process (GO:0000271) genes the transcript abundance for CS was found to be predominantly more in leaf samples, whereas, the transcript abundance for GAUT-like steadily increased from 65% to 90% and above from stage1 to stage5 indicating accumulation of galactomannan in developing seeds; and validated by qRT-PCR analysis. Galactomannan quantification by HPLC showed HG365 (12.98–20.66%) and HG870 (7.035–41.2%) gradually increasing from stage1 to stage 5 (10–50 DAA) and highest accumulation occurred in mature and dry seeds with 3.8 to 7.1 fold increase, respectively. This is the first report of transcriptome sequencing and complete profiling of guar seeds at different developmental stages, young pods, mature pods and young leaf material from two commercially important Indian varieties and elucidation of galactomannan biosynthesis pathway. It is envisaged that the data presented herein will be very useful for improvement of guar through biotechnological interventions in future.

## Introduction

Cluster bean or guar, *Cyamopsis tetragonoloba* (L) Taub (2n = 14) is an upright, coarse-growing large seeded, drought-tolerant, annual sub-tropical legume and a member of the *Leguminosae* (*Fabaceae*) family. Most of the improved varieties of guar have glabrous (smooth, not hairy) leaves, stems and pods. Pods are generally 1.6 to 4 inches long containing 5 to 12 seeds each. Seeds vary from dull-white to pink to light gray or black and range from 900 to 1,600 seeds oz^−1^. It is economically the most important of the four species in genus *Cyamopsis*. Guar is an important second largest cash crop of India accounting for over 80% of world production; it has tremendous application in textile, paper, petroleum, mining, pharmaceuticals, explosives, and food industries leading to earning of significant amounts of foreign exchange from export of guar splits, guar gum and several other products. Guar is believed to have originated in Africa but it has been grown throughout Southern Asia since ancient times as a vegetable and fodder crop. It is widely cultivated in countries like India, Pakistan, USA, Italy, Morocco, Germany, Greece, and Spain, thus considered as a new crop for western agricultural practices^1^. Galactomannan gum produced in the seed varies from 19–43% of the endosperm in various cultivated varieties of guar. India exported 4,94,101.27 metric tons of guar gum to the world worth 4,1695.60 Million INR or 646.94 Million USD during the years 2017–2018 http://apeda.gov.in/apedawebsite/SubHead_Products/Guargum.htm. Unlike seeds of some other legumes, guar has a large spherical-shaped endosperm which forms a viscous gel in cold water and is used as an emulsifier, thickener, and stabilizer in a wide range of foods and industrial applications. Guar gum is a Galactomannan, consisting of a linear (1 → 4)-β-linked D-mannan backbone with single-unit, (1 → 6) linked, α-D-galactopyranosyl side chains. Galactomannan are a heterogeneous group of cell wall storage polysaccharides and Galactomannan of most significant industrial use are obtained from the endosperms of legumes guar (*Cyamopsis tetragonoloba*) and locust bean (*Ceratonia siliqua*). Galactomannan serves as an excellent source of carbohydrate energy for developing seedling; assists drought avoidance before and during germination. It is deposited to the inside of primary cell walls of storage tissue. Galactomannan differ in their galactose content, with guar having a galactose to mannose ratio of approximately 1:2.

It grows well in soils of low fertility in the arid and semi-arid areas of the Tropics and Sub-Tropics where the rainfall is summer-dominant. In India, the sowing season for guar seed is end of July and it is harvested during November. It is usually 90–100 days crop. Guar is a rain fed monsoon crop. The World market for guar gum is estimated to be around 150,000 tons year^−1^, 70–80% of which is produced by India and Pakistan. USA is the largest consumer of guar gum with an annual consumption of 45,000 tons which represents 25% of world trade. Germany & Japan consume another 23% between them with the UK, Denmark and the Netherlands combined take further 22% of world trade. The world guar market is a mature one and increasing steadily (>2% per year).

Isolation of a cDNA clone encoding β-mannan synthase (ManS), that makes the β -1, 4-mannan backbone of Galactomannan belonging to a member of the cellulose synthase super gene family was reported by Dhugga *et al*.^2^ from guar seeds. The soybean somatic embryos expressing ManS cDNA contained high levels of ManS activities localized into Golgi was reported. Naoumkina *et al*.^3^ reported analysis of cDNA libraries from developing seeds of guar indicating that widely differing sets of genes are activated at the “early” and “late” developmental stages. Approximately 27% of the clones in the seed dataset correspond to novel proteins. The functional ontologies with the largest numbers of ESTs were signal transduction, carbohydrate metabolism, translation and protein processing. Metabolic and genetic perturbations accompany the modification of galactomannan in seeds of *Medicago truncatula* expressing mannan synthase from guar (*Cyamopsis tetragonoloba* L.) has been reported by Naoumkina *et al*.^4^.

Ng *et al*.^5^ reported an improved technique for measurement of Galactomannan in solutions by Differential Refractive Index (DRI) detector. Galactomannans do not dissolve completely into water; therefore, the actual dissolved concentration of the solution will always be ambiguous. Brahmi *et al*.^6^ reported genetic diversity in guar using allozyme polymorphism and a comparison was done with reported morphological diversity. The morphological data of 108 selected accessions was subjected to UPGMA clustering and fifty-five accessions were grouped based on allozyme polymorphism. Optimization and inference of PCR conditions for genetic variability studies of commercially important cluster bean varieties by RAPD analysis^7^ and molecular and morphological characterization of superior Cluster bean (C*yamopsis tetragonoloba*) varieties using molecular markers has been reported from my laboratory^8^. Genetic diversity study of cluster bean using RAPD and ISSR markers was reported^9^. Development of SCAR markers in cluster bean^10^. Development and validation of EST-derived SSR markers and genetic diversity analysis in cluster bean was reported^11^.

In the past various technologies like hybridization-based microarrays and Sanger sequencing based methods^12–14^ have been developed for transcriptome characterization of a population of cells. Of late high throughput sequencing based methods have opened new avenues for transcriptome based studies. It involves direct sequencing of cDNA, followed by mapping of the reads generated onto the genome. In recent times, NGS have been successful in offering fast and cost effective ways of studying genomic and transcriptome data^15^. With the the advent of various NGS technology platforms has opened new avenues in the field of functional genomics, gene identification and development of molecular markers^16^. With the help of transcriptome sequencing, thousands of genes can be identified and evaluated in a single analysis^17^. The availability of RNA-seq data of many model and non model plants has led to the rapid development of EST libraries^18–21^ and molecular markers like SSRs and SNPs in a cost effective manner has been reported^22,23^.

The construction of cDNA libraries using RNA isolated from developing endosperms at four different development stages of active accumulation between 25 to 38 days post anthesis of Galactomannan biosynthesis in fenugreek (*Trigonella foenum-graecum*) seeds has been reported by Wang *et al*.^24^. DNA from these libraries was sequenced using the 454 sequencing technology to yield a total of 1.5 million expressed sequence tags (ESTs). RNAseq of leaf tissues derived from 3-week-old field grown plants of guar varieties M-83 and RGC-1066 was recently reported by Tanwar *et al*.^25^. 62,146 non-redundant unigenes with an average length of 679 bp were reported. A total of 11,308 guar unigenes were annotated and grouped into six categories having 55 subclasses along with identification of SSR and SNP markers. Kaila *et al*.^26^ reported that cluster bean variety RGC-936 has a quadripartite structured chloroplast genome with a large single copy of 83,025 bp and small single copy of 17,879 bp having total size of 152,530 bp and shows similar organization to other closely related legumes but differs in inverted repeat region. Transcriptome sequencing of leaf, shoot and flower derived from cluster bean variety RGC-936 with 48,007 unigenes^27^. Tyagi *et al*.^28^ reported miRNA sequencing of leaf, stem, root, bud and seed derived from cluster bean variety RGC-936 and its association with galactomannan biosynthesis. Keeping in view the economic importance and industrial applications of guar gum and availability of very little information regarding comprehensive transcriptome profiling of developing seeds of cluster bean; therefore, present investigation of transcriptome sequencing of seeds collected at different developmental stages (days after anthesis), pods young, pods mature and leaf samples (fourteen in all) of commercially important Indian varieties of cluster bean for elucidation of galactomannan biosynthesis pathway genes was undertaken.

## Results and Discussion

### De novo transcriptome assembly

The data comprising of 100 bp paired end raw reads was imported in CLC Work Bench and subjected to base quality trimmed QC and clean data with average PHRED score of 60 to 65 were merged and subjected to *de novo* assembly. Fourteen processed clean data read files from seven developmental stages (stage1 to stage7) of two guar varities were incorporated in the *de novo* assembly to produce 1,13,607 contiguous sequences (contigs). Contig sizes ranged from 86 to 17,022 bases with an N50 of 1,244 bases as shown in Supplementary Figure 1.

### Functional annotation and analysis of unigenes

**Figure 1:**
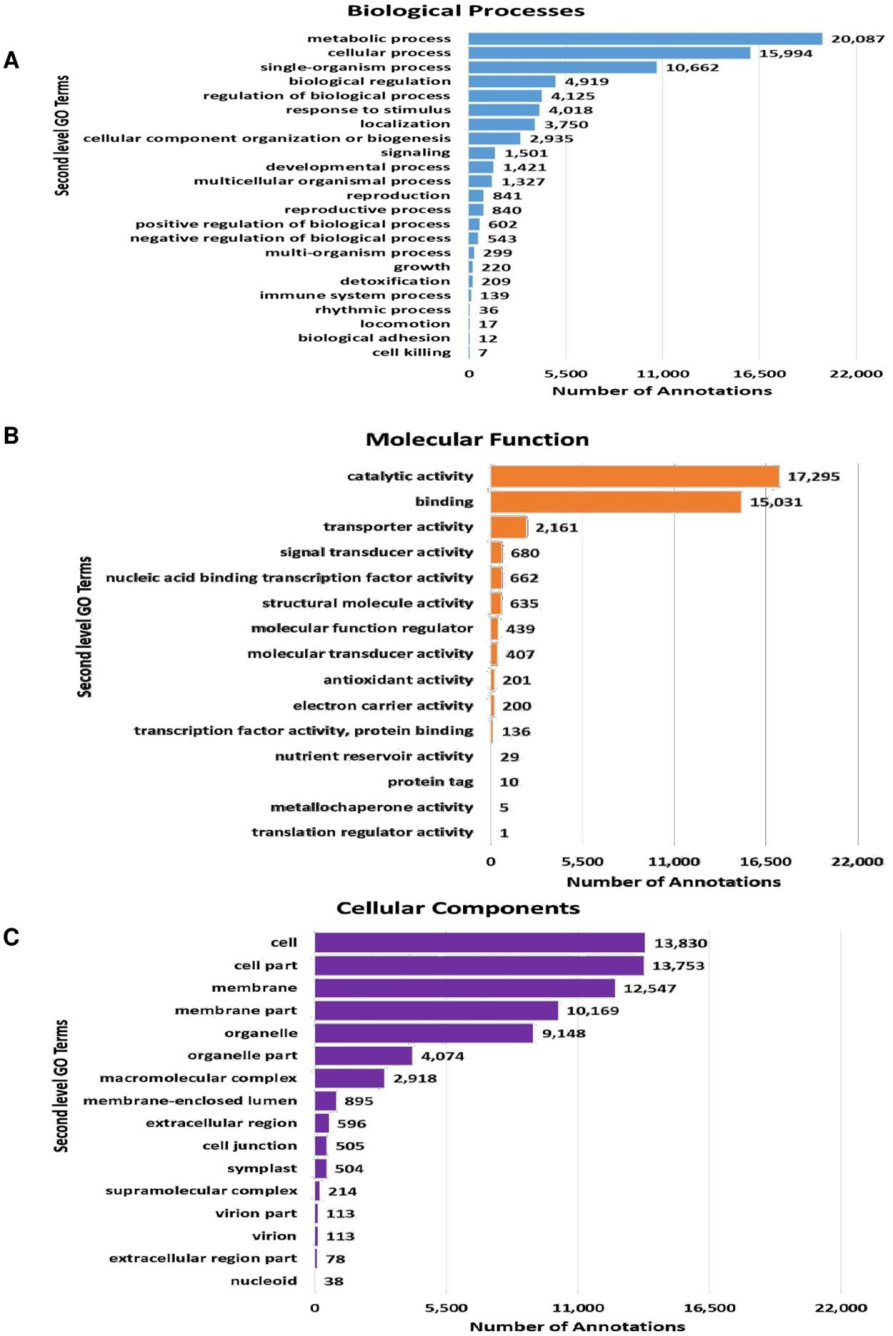
Second level Gene Ontology (GO) term functional classification assigned to *Cyamopsis tetragonoloba* contigs assembled from all sequencing reads according to (**A**) Biological Process; (**B**) Molecular Functions and (**C**) Cellular Components.

The sequence annotation statistics reveal that InterProScan Ids were assigned to all the 1,13,607 contigs, out of which 43,133 contigs showed Blast hits, 37,039 contigs were mapped and 30,134 contigs were completely annotated with GO mapping and InterProScan respectively as shown in Supplementary Figure 1. Top hit species distribution analysis showed that guar unigenes have a very high homology to several other legume species such as, *Glycine max* and *Glycine soja* followed by *Medicago truncatula* and *Vigna radiata* as shown in Supplementary Figure 1D. The BLASTX program was used for the annotation of assembled unigenes from pods young and pods mature against the Nr database, with a set cut off *E*-value of 1e^−5^ and the percent base match for top hit homology identification are shown in Supplementary Figure 1E,F. On the basis of homology results, the unigenes were assigned different GO terms. The GO terms were distributed into functional groups based on BLASTX results, which were further classified into three main categories viz. Biological Processes, Molecular Functions and Cellular Components as shown in Fig. 1. The three gene ontologies are related in a parent child hierarchy wherein at higher levels parental ontologies govern the sownstream processes. For example if a contig has a GO level of 6, there are five parent levels above with the child level being more discrete than the parent levels. Contigs comprising the more discrete child GO terms were analysed for differential expression in comparison between the HG870 and HG365 varieties. The top GO terms in Biological Processes category was found to be Metabolic process” (20,087), “Cellular process” (15,994), “Single-organism process” (10,662) and “Biological regulation” (4,919). Similarly, the major GO terms observed in Molecular Functions category were “Catalytic activity” (17,295), “Binding” (15,031) and “Transporter activity” (2161) and Signal transducer activity (680), whereas, in Cellular Componenets category GO terms like Cell (13,830), Cell part (13,753), Membrane (12,547), Membrane part (10,169) were highly enriched. Our results of functional annotation and analysis of unigenes are in agreement to the data reported for leaf, shoot and flower^27^ from guar variety RGC-936 and leaf^25^ of guar variety RGC-1066 and M-83.

### Gene expression analysis (DEG) and enzyme discovery

**Figure 2:**
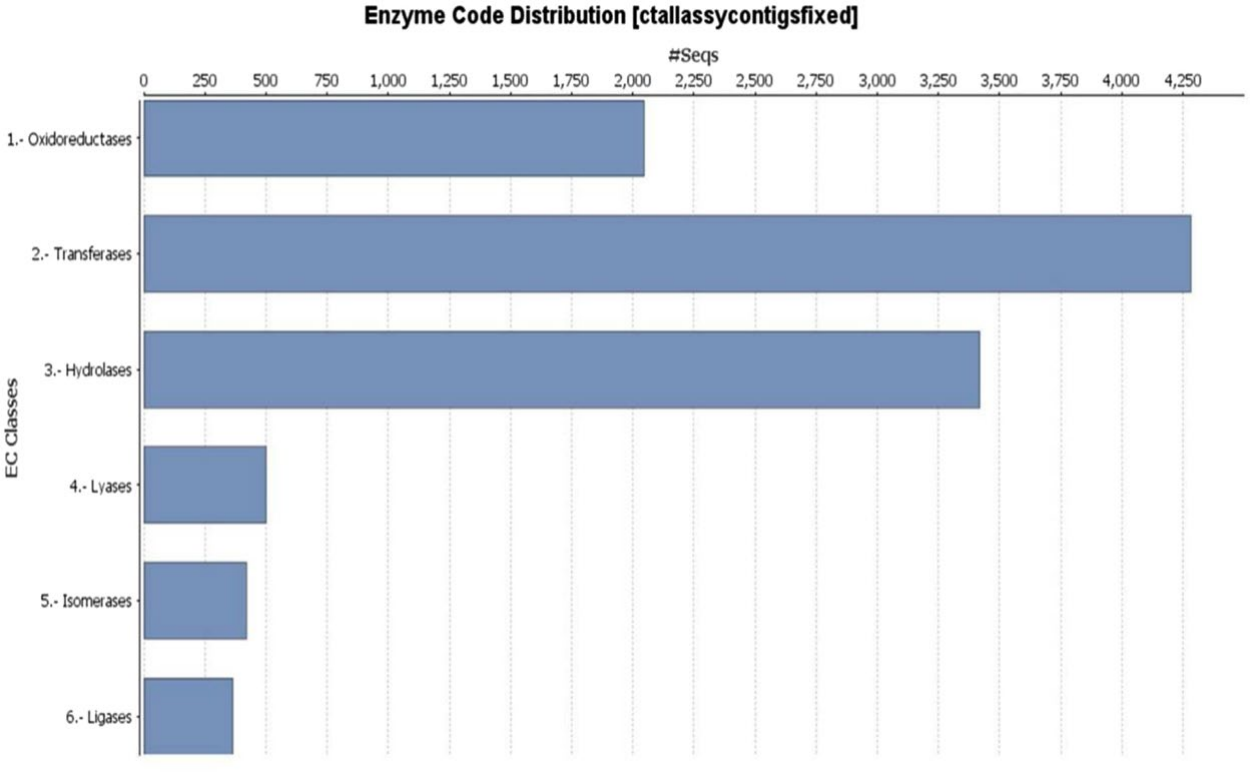
Enzyme code (E.C.) class distribution among annotated contigs of *Cyamopsis tetragonoloba*.

Among the total number of 43,133 contigs that showed Blast hits, 37,039 contigs were mapped and 30,134 contigs were completely annotated and thereby assigned Enzyme Codes (EC). All the unigenes assigned with EC numbers were grouped into six different classes of enzymes, with transferases, hydrolases and oxidoreductases accounting for 89.8% the major enzyme classes as shown in Fig. 2. Predicted enzyme code distribution showed (Fig. 2); about 4,300 enzymes with transferase capabilities, 3,375 with hydrolase activity and more than 2,000 enymes with oxidoreductase activity. Lyase, isomerase and ligases comprised the remaining three enyme class with each category comprising of 511 contigs or less accounting for 10.2% of the reported enzyme assignments.

**Figure 3:**
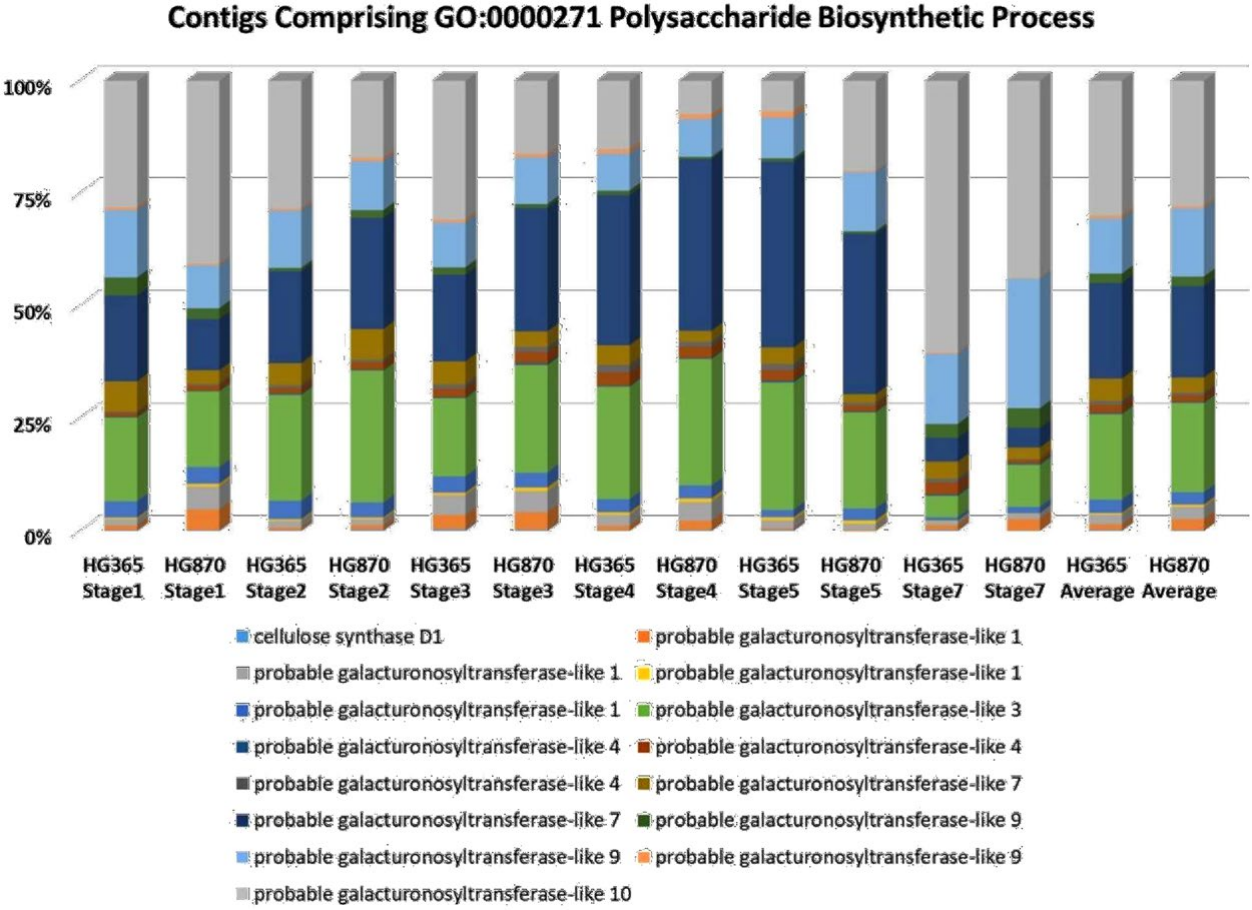
Percentages of contigs assigned to GO:0000271 Polysaccharide Biosynthetic Process of *Cyamopsis tetragonoloba* contigs assembled from all sequencing reads.

Gene ontology studies of the transcriptome data identified major genes involved in polysaccharide biosynthesis such as Cellulose Synthase D1 (CS D1) and Galacturonosyltransferase -like (GAUT -like) gene families. Among the polysaccharide biosynthesis (GO:0000271) genes, the transcript abundance was shown as percentage of contigs present in the corresponding gene family as shown in Fig. 3. Further, we studied the expression pattern of CS D1 gene and GAUT-like gene family based on RPKM values at different developmental stages1–5 and stage7 (young leaf) in HG365 and HG870 varieties of guar (Fig. 3). Among the polysaccharide biosynthetic process (GO:0000271) genes the transcript abundance for CS D1 was found to be predominantly more in leaf samples stage7, whereas, the transcript abundance for GAUT-like steadily increased from 65% to 90% and above from stage1 to stage5 indicating accumulation of galactomannan in developing seeds. Among the two varieties the transcript abundance for GAUT-like were consistently higher in HG870 as compared to HG365. Therefore, higher expression of GAUT -like family genes during seed development is directly linked to accumulation of galactomannan in guar seeds (highest being in stage5). Our results are in agreement to the data reported for transcript abundance for accumulation of galactomannan in developing seeds from 25 to 38 days post anthesis in fenugreek^24^.

**Figure 4:**
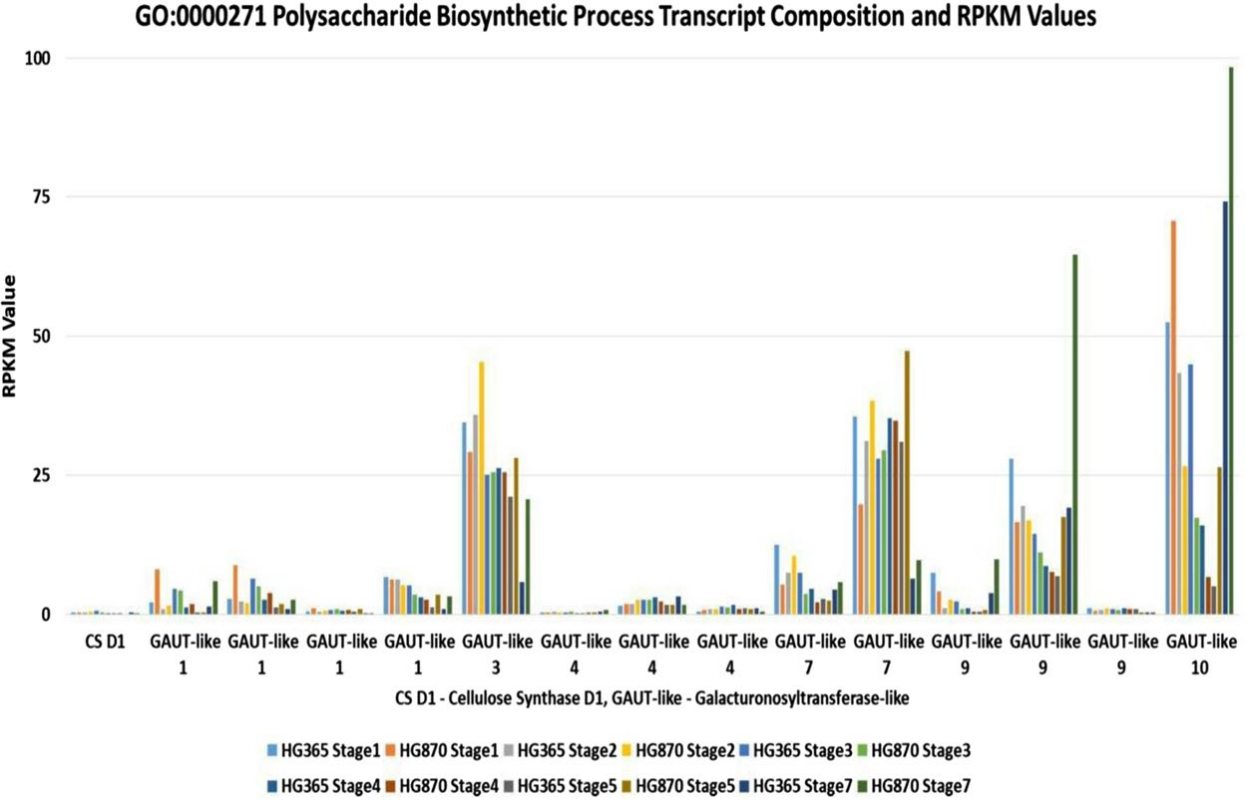
Trends of the relative expression of identified GO:0000271 Polysaccharide Biosynthetic Process of *Cyamopsis tetragonoloba* contigs assembled from all sequencing reads.

**Figure 5:**
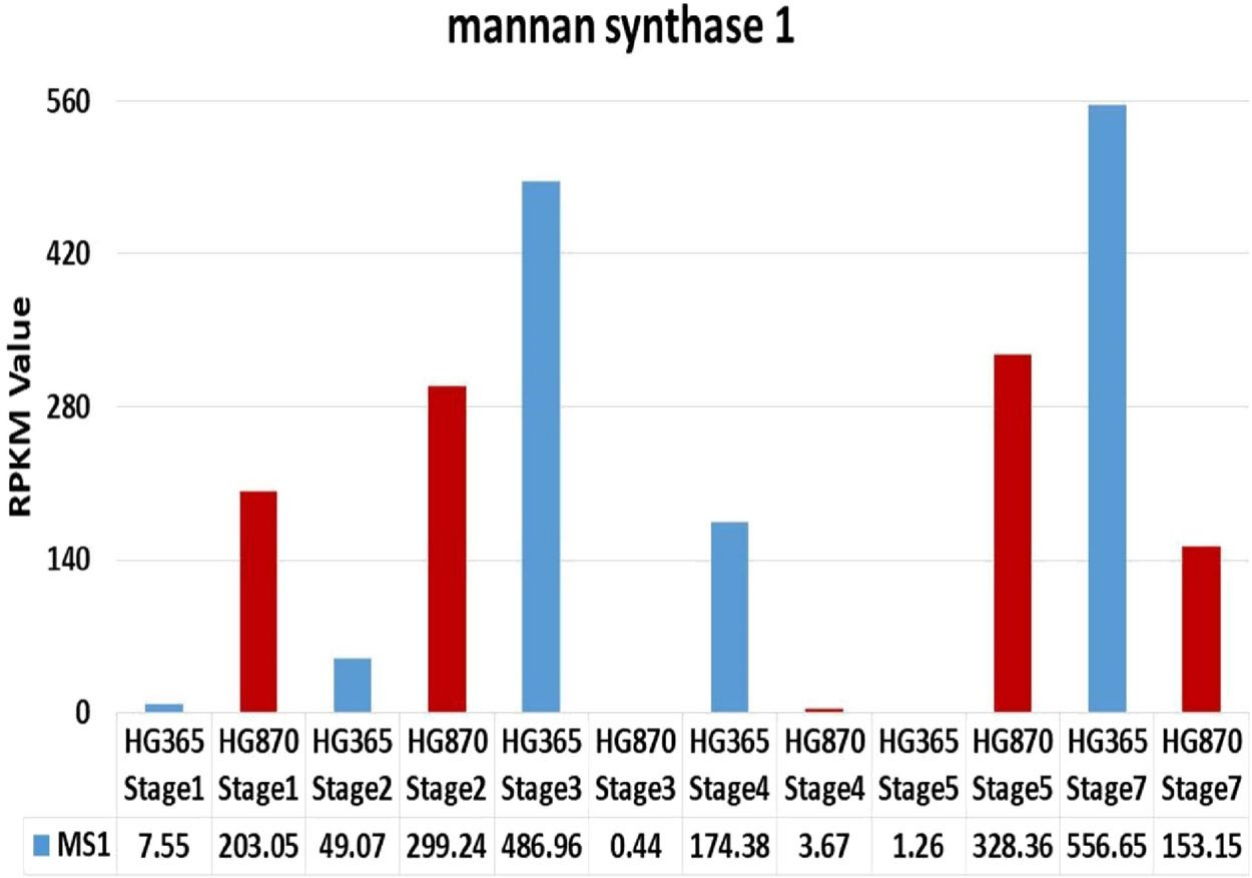
Relative expression of identified Mannan synthase 1 of *Cyamopsis tetragonoloba* contigs assembled from all sequencing reads.

Similarly, relative expression pattern of CS D1 and GAUT –like (1,3,4,7,9,10) gene families based on RPKM values in two varieties HG365 and HG870 was also found to be developmentaly regulated from stage1 to stage5 indicating accumulation of galactomannan in developing seeds as shown in Fig. 4. The relative expression profile of three other important enzymes involved in Galactomannan biosynthesis based on RPKM values derived from transcriptome data showed that Mannan synthase-1 (MS-1) (Fig. 5), Xyloglucan glycosyltransferase isoforms (CSLC) (Supplementary Figure 2) and Galactinol-sucrose galactosyltransferase-2 (AGA2) (Supplementary Figure 3) also showed similar trend. Among the above 3 genes the expression level (RPKM) of MS-1 and AGA2 has shown to increase gradually till the later stages of pod development which is directly linked to biosynthesis and storage of galactomannan in guar seeds. The KEGG pathway enrichment of DEG’s for GO:0051070 Galactomannan biosynthesis process has been presented as Supplementary Figure 4.

### Validation of PCR primers

**Figure 6:**
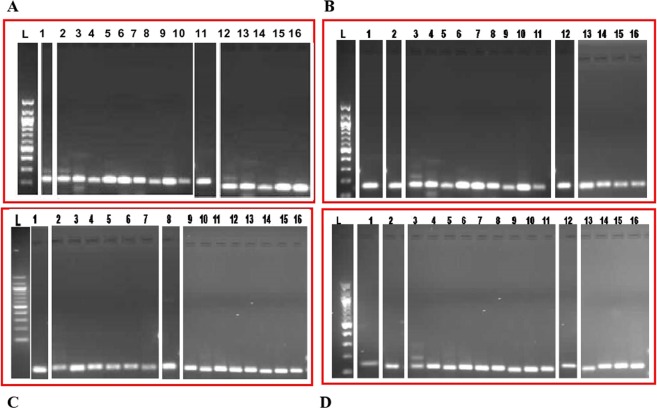
Validation of PCR primers for gene families or enzymes involved in Galactomannan biosynthesis from two elite commercially important Indian Guar (*Cyamopsis tetragonoloba* L) varieties, namely, HG365 (lanes marked as 1–8) and HG870 (lanes marked as 9–16) at different developmental stages; Days After Anthesis (DAA) e.g. 10, 20, 30, 40 and 50, leaf, pods young, pods mature. L denotes 100 bp ladder. (**A**) Primer Code 3 representing Ct_9050; Hexokinase-1 (152 bp); (**B**) Primer Code 10 representing Ct_2106; Sucrose synthase (152 bp); (**C**) Primer Code 11 representing Ct_12647; Mannan synthase (144 bp); (**D**) Primer Code 13 representing Ct_1088; Alpha galactosidase (150 bp). The images for this figure have been cropped and full length gel pictures have been shown as Supplementary Figure 8.

Validation of PCR primers for gene families or enzymes involved in Galactomannan biosynthesis from two elite commercially important Indian Guar (*Cyamopsis tetragonoloba* L) varieties, namely, HG365 and HG870 was performed by designing twenty two sets of forward and reverse primers as shown in Table 1. PCR amplification of cDNA obtained from mRNA isolated from pods collected from guar varieties HG365 and HG870 at different developmental stages; Days After Anthesis (DAA) e.g. 10, 20, 30, 40 and 50; pods young, pods mature and young leaves showed a variable response as shown in Table 2. Experiments were repeated twice with separate isolation of RNA from all the samples, synthesis of cDNA and PCR amplification for ruling out experimental errors. The Ct_9050; Hexokinase, Primer Code 3 showed amplicon size of 152 bp at all the stages of development as shown in Fig. 6A. The Ct_2106; Sucrose synthase, Primer Code 10 showed amplicon size of 152 bp at all the stages of development for both the varieties as shown in Fig. 6B. The Ct_12647; Mannan synthase, Primer Code 11 showed amplicon size of 144 bp at all the stages of development for both the varieties as shown in Fig. 6C. The Ct_1088; Alpha galactosidase, Primer Code 13 showed amplicon size of 150 bp at all the stages of development for both the varieties as shown in Fig. 6D. Validation of twenty two sets of PCR primers for gene families or enzymes involved in Galactomannan biosynthesis from two elite commercially important Indian guar (*Cyamopsis tetragonoloba* L) varieties, namely, HG365 and HG870 have been summarized in Table 2 with either amplification of desired size amplicon (S), or more than one band (M) or no amplification (N).

**Table 1:** Real Time qPCR/PCR Primers for gene families or enzymes involved in Galactomannan biosynthesis from two elite commercially important Indian Guar (*Cyamopsis tetragonoloba* L) varieties, namely, HG365 and HG870.

Primer Code	Lab designation	Enzyme Name	EC No.	Forward Primer 5′ to 3′	Reverse Primer 5′ to 3′	Amplicon size bp	Predicted T_m_ value (°C)
1	Invertase – Ct_27403	Acid beta-fructofuranosidase	3.2.1.26	AGCTTTGCACAAGGTGGAAG	TGCAGAATTCATCTGCCAAA	147	60.43 and 60.34
2	Invertase – Ct_4555	Acid beta-fructofuranosidase	3.2.1.26	GGCGAAAAACTGTCCTTGAG	GCCTCAGTTGCATTGTTGAA	152	Both – 59.85
3	Hexokinase – Ct_9050	hexokinase-1-like isoform X3 [Cucumis melo]	2.7.1.1	GGGAGGAGCAGAAGTCAGTG	GCCTGAGCCATCATTAGCAT	152	59.99 and 60.21
4	Hexokinase – Ct_10978	hexokinase- chloroplastic	2.7.1.1	CGATTTACAAGCCGTTGGAT	TTCTGCAAAATCCCCACAAT	150	59.96 and 60.31
5	Hexokinase – Ct_601	hexokinase [Medicago truncatula]	2.7.1.1	CGGGGAGAAACAAAAATCAG	AATTCCAGAGCCGTCATTTG	157	59.54 and 60.07
6	Phosphomannomutase 2 – Ct_11030	phosphomannomutase [Vigna radiata radiata]	5.4.2.8	TGCAGCCAAGAAGAAAGAGA	CCCAACCTTGAGGAAAAACA	157	58.87 and 59.94
7	Phosphomannoisomerase – Ct_4332	Mannose-6-phosphate isomerase [Glycine soja]	5.3.1.8	GGTTCTGAGGTTGGAAAAGC	CATTCAATGCACTCACCACA	147	58.77 and 59.09
8	Sucrose Synthase – Ct_154	sucrose synthase	2.4.1.13	TGCCAACATTTGCTACTTGC	GGTCCCAGTGAGAAGGATCA	153	59.88 and 60.05
9	Sucrose Synthase – Ct_3680	sucrose synthase 2	2.4.1.13	AGGCCATGACTTGTGGACTC	CCTTGCATCGTTGGAAGAAT	150	60.12 and 60.07
10	Sucrose Synthase – Ct_2106	sucrose synthase [Glycine max]	2.4.1.13	TGTAATGGTGGTCCTGCTGA	CCGCCCTGAGAGATTTTGT	152	60.11 and 60.20
11	Mannan Synthase – Ct_12647	Glucomannan 4 beta mannosyl transferase mannan synthase 1	2.4.1.32	AGCAGATGCTGCCAAGAAAT	TTGTGCTTCCCATGAACAAA	144	59.98 and 60.09
12	Mannan Synthase – Ct_20538	Glucomannan 4 beta mannosyl transferase mannan synthase 1	2.4.1.32	TGGAAATGCCATGAAACAGA	GGTCGTGTCCGAATAACAGG	146	60.05 and 60.38
13	Alpha galactosidase – Ct_1088	alpha-galactosidase [Medicago truncatula]	3.2.1.22	TGAGATATGGGCAGGTCCAC	TTGTGCTCCCAAAGGTCTCT	150	60.89 and 59.84
14	UDP-galactose 4-epimerase – Ct_16689	UDP-glucose 4-epimerase [Medicago truncatula]	5.1.3.2	CTGGTCGTGGTACGTCAGTG	TTTGCCTTCCAACCAAGTTC	157	60.22 and 60.09
15	Fructokinase – Ct_10145	fructokinase-4	2.7.1.4	GGATGCTGTTGACACCACTG	TTCACAGTCAATGCACCACA	147	60.16 and 59.71
16	Fructokinase – Ct_41766	fructokinase-5	2.7.1.4	TGGTGCTGGTGACTCTTTTG	CAGGAATTGCTCCCTTCTGT	149	59.87 and 59.28
17	Fructokinase – Ct_23710	fructokinase-7 isoform 1 [Glycine max]	2.7.1.4	TGCATTTGTCAGTGGCATTC	AGTGCAGGAATTGCACCTTT	141	60.68 and 59.74
18	Fructokinase – Ct_68360	fructokinase-7	2.7.1.4	ATGCATTTGTTGGTGGGATT	TGTAGGCAGTGCAGGAATTG	149	60.06 and 59.86
19	GDP-mannose pyrophophorylase (GMP) – Ct_3368	Glucose-1-phosphate adenylyltransferase family isoform 1 [Theobroma cacao]	2.7.7.22	AGGTGTCAGGCTTTCACGTT	TGGACGTCTTCTCCAAGGAT	147	59.77 and 59.65
20	GDP-mannose pyrophophorylase (GMP) – Ct_2178	mannose-1-phosphate guanylyltransferase 1 [Eucalyptus grandis]	2.7.7.22	TGTGGGAAATGTCATTGTGG	GCAAGCGTGCTTCTTAATCC	157	60.22 and 59.99
21	Galactosyl transferase* (GalT) – Ct_16574	Galactosyl transferase GMA12 MNN10 family isoform 1 [Theobroma cacao]	2.4.1.22	ACGGGGTGTCAACCTTGTAG	CGGCGAAACGGAATTATCTA	156	59.88 and 60.05
22	Galactosyl transferase ^*^ (GalT) – Ct_18788	galactosyl transferase GMA12 MNN10 family [Medicago truncatula]	2.4.1.22	TGATGAGGTGGAGAGGAAGG	GCCATTACAGGGTTGACACC	151	60.19 and 60.24

^*^Galactosyl transferase GMA12 MNN10 family [*Medicago truncatula*] – there are no known plant enzymes with the 2.4.1.22 EC assigned to them. The galactosyl transferase contigs were aligned to the *M. truncatula* XM_013602472 annotation shown above. Best hit was to: 1. Q564G7 (GMGT1_CYATE) Galactomannan galactosyltransferase 1 OS = *Cyamopsis tetragonoloba* GN = GMGT1 PE = 1 SV = 1 Length = 435 - Identities = 435/435 (100%).

**Table 2:** Validation of twenty two sets of PCR primers for gene families or enzymes involved in Galactomannan biosynthesis from two elite commercially important Indian guar (*Cyamopsis tetragonoloba* L) varieties, namely, HG365 and HG870. PCR amplification of cDNA obtained from RNA isolated from pods collected from guar varieties at different developmental stages; Days After Anthesis (DAA) e.g. 10, 20, 30, 40 and 50; young leaves from germinated seedlings, pods young (immature), pods mature; represented by Stage1, Stage2, Stage3, Stage4, Stage5, Stage7, PY and PM, respectively.

		HG365								HG870								
Lab Designation	Annotation (bp)	Stage 1	Stage 2	Stage 3	Stage 4	Stage 5	Stage 7	PY	PM	Stage 1	Stage 2	Stage 3	Stage 4	Stage 5	Stage 7	PY	PM	Primer code
Ct_27403	Acid beta-fructofuranosidase 147 bp	S	S	S	S	S	S	S	S	S	S	S	S	N	S	S	S	**1**
Ct_4555	Acid beta-fructofuranosidase 152 bp	S	S	S	S	S	S	M	M	S	S	S	M	M	S	S	M	**2**
Ct_9050	*Hexokinase-1-like isoform X3 [Cucumis melo] 152 bp*	S	S	S	S	S	S	S	S	S	S	S	S	S	S	S	S	**3**
Ct_10978	Hexokinase- chloroplastic 150 bp	N	M	N	N	S	S	N	S	S	N	S	M	S	N	S	N	**4**
Ct_601	*Hexokinase [Medicago truncatula] 157 bp*	S	M	M	M	M	N	M	M	S	M	S	M	S	S	S	S	**5**
Ct_11030	*Phosphomannomutase [Vigna radiata radiata] 157 bp*	S	S	M	S	S	S	S	M	S	S	M	N	S	M	S	S	**6**
Ct_4332	*Mannose-6-phosphate isomerase [Glycine soja] 147 bp*	S	S	M	M	S	S	S	S	S	S	M	N	M	N	S	S	**7**
Ct_154	Sucrose synthase 153 bp	S	S	M	S	S	N	S	S	S	S	M	S	M	M	S	S	**8**
Ct_3680	Sucrose synthase 2 150 bp	S	S	S	S	S	M	S	S	S	S	S	S	M	S	S	S	**9**
Ct_2106	*Sucrose synthase [Glycine max] 152 bp*	S	S	S	S	S	S	S	S	S	S	S	S	S	S	S	S	**10**
Ct_12647	Mannan synthase 144 bp	S	S	S	S	S	S	S	S	S	S	S	S	S	S	S	S	**11**
Ct_20538	Mannan synthase 1 146 bp	M	S	S	S	S	S	S	M	S	M	S	S	S	N	S	S	**12**
Ct_1088	*Alpha-galactosidase [Medicago truncatula] 150 bp*	S	S	S	S	S	S	S	S	S	S	S	S	S	S	S	S	**13**
Ct_16689	*UDP-glucose 4-epimerase [Medicago truncatula] 157 bp*	N	M	M	N	S	S	S	M	M	M	S	M	N	S	S	S	**14**
Ct_10145	Fructokinase-4 147 bp	S	S	N	S	S	S	S	M	S	M	S	N	S	N	M	M	**15**
Ct_41766	Fructokinase-5 149 bp	M	M	N	S	S	S	S	M	M	S	S	S	M	N	M	S	**16**
Ct_23710	*Fructokinase-7 isoform 1 [Glycine max] 141 bp*	S	M	M	S	S	S	S	S	S	M	S	S	M	S	S	M	**17**
Ct_68360	Fructokinase-7 149 bp	M	M	M	S	S	N	S	S	M	M	S	M	M	M	M	S	**18**
Ct_3368	*Glucose-1-phosphate adenylyltransferase family isoform 1 [Theobroma cacao] 147 bp*	M	S	M	S	S	N	S	S	S	S	S	S	S	S	S	S	**19**
Ct_2178	*Mannose-1-phosphate guanylyltransferase 1 [Eucalyptus grandis] 157 bp*	S	S	M	S	S	S	S	S	S	S	S	S	M	N	S	S	**20**
Ct_16574	*Galactosyl transferase GMA12 MNN10 family isoform 1 [Theobroma cacao] 156 bp*	S	S	M	S	S	M	S	S	S	S	S	S	S	S	S	S	**21**
Ct_18788	*Galactosyl transferase GMA12 MNN10 family [Medicago truncatula] 151 bp*	S	S	M	S	S	S	S	S	S	S	S	S	S	S	S	S	**22**

S-Desired Single amplicon; M- More than one amplicon; N- No amplification.

### Fold change expression analysis by qRT-PCR

The average CT values of the three housekeeping genes SPT5, HPATcp and PAGM are shown in Supplementary Figure 5. It was found that SPT5 and HPATcp gave fairly high CT values and lot of inconsistency (unstable), whereas, PAGM gave fairly low and stable average CT values across different developmental stages of pod/seed formation employed from stages 1 to 8. Therefore, PAGM was chosen as the most stable housekeeping gene for normalizing the data. The specificity of primers employed in the present investigation for PAGM as a house keeping gene across eight developmental stages of pod/seed formation, leaf, pods young, pods mature in qRT-PCR analysis as Melt curve with all stage samples showing single peak is shown in Supplementary Figure 6. Similarly, the specificity of primers for the enzymes involved in galactomannan biosynthesis; e.g. gene of interest Ct_2106; Sucrose synthase, Primer Code 10 (152 bp) Ct_12647 and Mannan synthase, Primer Code 11(144 bp) across eight developmental stages of pod/seed formation, leaf, pods young, pods mature in qRT-PCR analysis as Melt curve with all stage samples showing single peak is shown in Supplementary Figure 7. Similar results of Melt curve were obtained for other primer code enzymes involved in galactomannan biosynthesis in the present investigation (data not shown).

**Figure 7:**
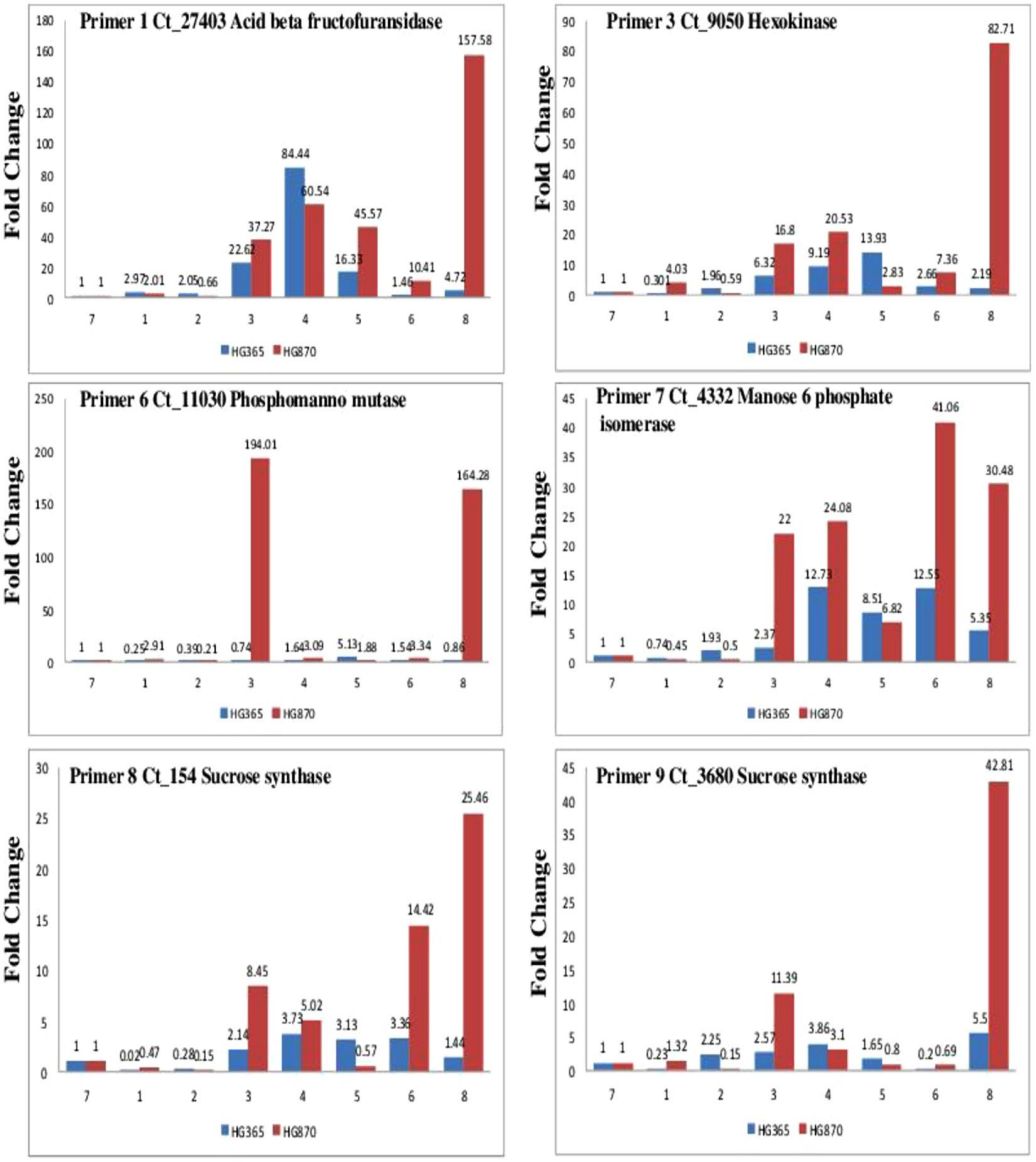
Gene expression profile in terms of fold change of Primer codes 1, 3, 6, 7, 8, and 9 represented by Acid beta fructofuranosidase (Invertase), Hexokinase, Phosphomannomutase, mannose-6-phosphate isomerase, and sucrose synthase for genes of interest involved in Galactomannan biosynthesis from two elite commercially important Indian Guar (*Cyamopsis tetragonoloba* L) varieties, namely, HG365 and HG870 at different developmental stages; Days After Anthesis (DAA) e.g. 10, 20, 30, 40 and 50 leaf, pods young, pods mature (denoted by numerals 1–8).

**Figure 8:**
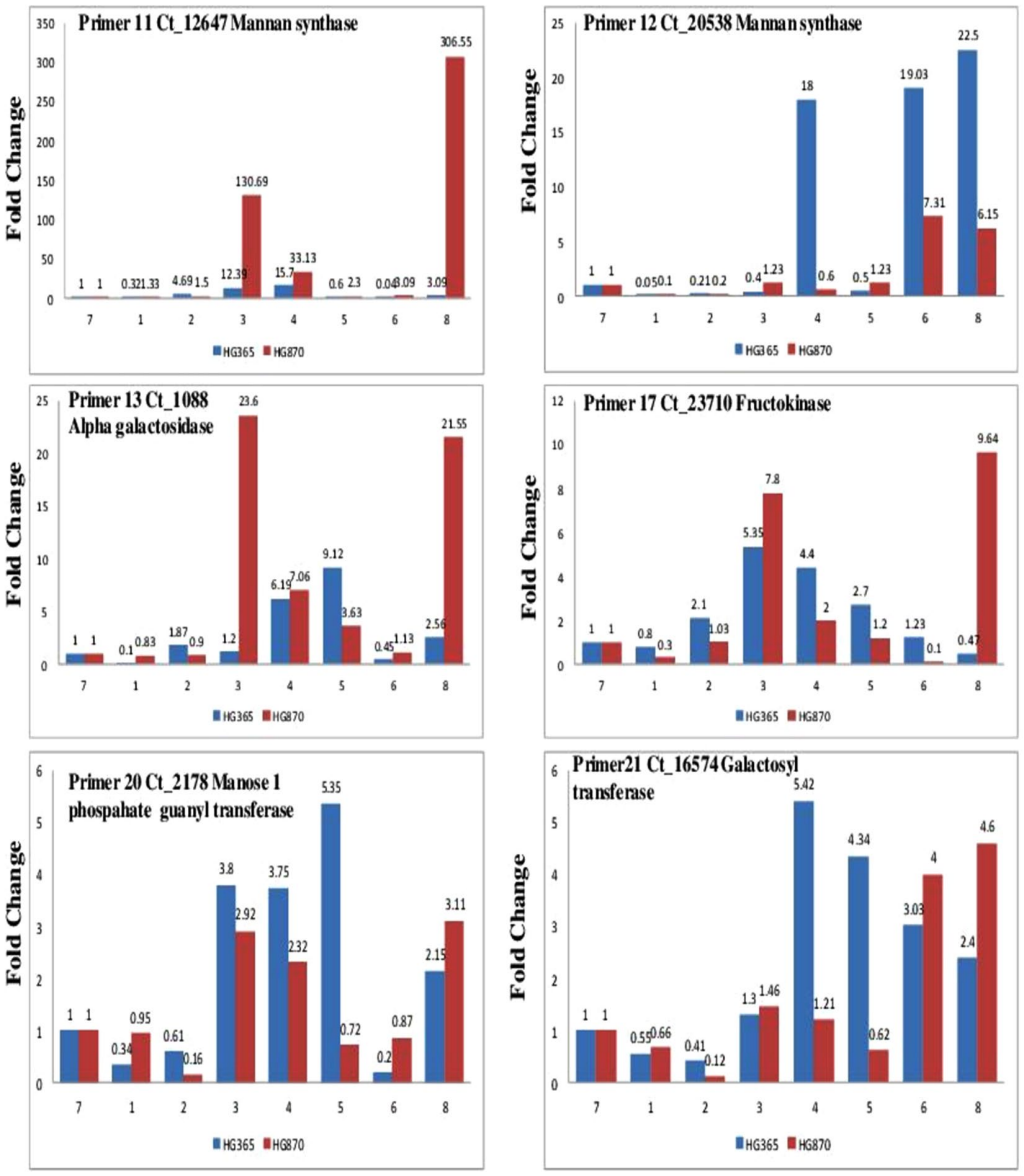
Gene expression profile in terms of fold change of primer codes 11, 12, 13, 17, 20 and 21 represented by Mannan synthase, Alpha galactosidase, Fructokinase, Manose 1 phosphate guanyl transferase and Galctosyl transferase for genes of interest involved in Galactomannan biosynthesis from two elite commercially important Indian Guar (*Cyamopsis tetragonoloba* L) varieties, namely, HG365 and HG870 at different developmental stages; Days After Anthesis (DAA) e.g. 10, 20, 30, 40 and 50 leaf, pods young, pods mature (denoted by numerals 1–8).

Fold change gene expression profile of Primer codes 1, 3, 6, 7, 8, and 9 represented by Acid beta fructofuranosidase (Invertase), Hexokinase, Phosphomannomutase, mannose-6-phosphate isomerase, and sucrose synthase are shown in Fig. 7 and primer codes 11, 12, 13, 17, 20 and 21 are shown in Fig. 8 represented by Mannan synthase, Alpha galactosidase, Fructokinase, Manose 1 phosphate guanyl transferase, Galctosyl transferase involved in Galactomannan biosynthesis of two cluster bean varieties HG365 and HG870 derived samples collected at different developmental stages of pod/seed formation (DAA) Days After Anthesis e.g. 10, 20, 30, 40, 50, leaf, young pods and mature pods (represented by numbers 1, 2, 3, 4, 5, 6, 7, and 8) was achieved. The expression profile in HG870 has been found to be higher across all stages of development from stage 1, 2, 3, 4, 5, 6 and 8 as compared to stage 7 of young pods used as a calibrator. Several of the enzymes such as Invertase, Hexokinase, Phosphomannomutase, mannose-6-phosphate isomerase, sucrose synthase, and mannan synthase were found to show low fold change gene expression in early stages (1, 2) of development which gradually increased from stage 3 to 5 and in many cases was found to be highest in stage 8 at mature stage. Indicating thereby that these enzymes are specifically involved in galactomannan biosynthesis with highest accumulation and expression in stage 8 the mature pod. However, Stage 6 representing leaf samples consistently showed low fold change gene expression in all the primers except for primer 7 mannose-6-phosphate isomerase, primer 8 sucrose synthase, primer 12 mannan synthase and primer 21 galactosyl transferase which indicates that these enzymes are actively expressed in leaf tissues. Our results of Mannan synthase expression profile are in agreement to the data presented for guar variety RGC-936 seed^28^ miRNA transcriptome. This data of high gene expression of galactomannan biosynthesis in cluster bean mature pods was further validated by performing quantification of galactomannan in various stages of seed development, endosperm and mature seed samples.

### Quantification of galactomannan

**Figure 9:**
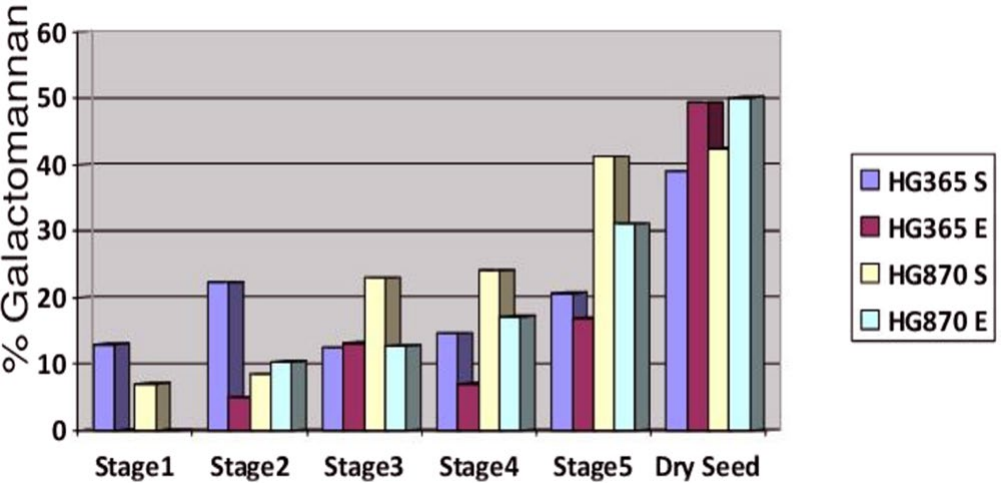
Percentage Galactomannan content in Alcohol Insoluble Residues obtained from two elite commercially important Indian guar (*Cyamopsis tetragonoloba* L) varieties, namely, HG365 and HG870 through HPLC. Seeds from pods collected from guar varieties at different developmental stages; Days After Anthesis (DAA) e.g. 10, 20, 30, 40 and 50; dry seeds; represented as HG365S and HG870S. Endosperm of seeds from pods collected from guar varieties at different developmental stages; Days After Anthesis (DAA) e.g. 20, 30, 40 and 50; and endosperm powder represented as HG365E and HG870E derived from dry seeds.

The seeds samples at different developmental stages, namely, Stage 1, 2, 3, 4, 5 and dry seeds and endosperm of stage 2, 3, 4, 5, dry seeds and seed coat were employed for quantification of galactomannan in alcohol insoluble residues as shown in Fig. 9. The HPLC analysis of alcohol insoluble residue extracts of HG365 seeds revealed that galactomannan content varied from 12.98% to 20.66% across Stage1 to Stage5 (10 to 50 days post anthesis) and HG365 dry seed extracts exhibited 38.95% galactomannan content. Similarly, extracts of HG365 endosperm revealed that galactomannan content varied from 4.86% to 16.95% across Stage2 to Stage5 and HG 365 dry seed endosperm extracts exhibited 49.375% galactomannan content as shown in Fig. 9. On the other hand, extracts derived from HG870 seeds revealed that galactomannan content varied from 7.035% to 41.2% across Stage1 to Stage5 and HG870 dry seed extracts exhibited 42.49% galactomannan content. Similarly, extracts of HG870 endosperm revealed that galactomannan content varied from 10.325% to 31.105% across Stage2 to Stage5 (10 to 50 days post anthesis) and HG870 dry seed endosperm extracts exhibited 50.145% galactomannan content as shown in Fig. 9. Overall the galactomannan content in both the varieties HG365 and HG870 gradually increased from Stage1 through Stage5 (10 to 50 days post anthesis) and highest accumulation occurred in mature and dry seeds with 3.8 to 7.1 fold increases, respectively. Guar variety HG870 showed higher accumulation of galactomannan content over HG365. The extract derived from seed coat of both the varieties exhibited negligible amount of galactomannan content. Low levels of glucose and fructose varying from 0.01% to 0.08% for HG365 variety and 1.34% to 3.48% for HG870 variety was obtained in early stages of seed development of Stage2, Stage3 and Stage4 (20, 30, 40 days after Anthesis; data not shown). Similarly, accumulation of various sugars and deposition of galactomannan in endosperm has been reported across various stages of seed development from 25 to 50 days post anthesis in *Trigonella foenum graceum*^24^ confirming that they are developmentally regulated.

### Proposed galactomannan biosynthesis pathway in cluster bean

**Figure 10:**
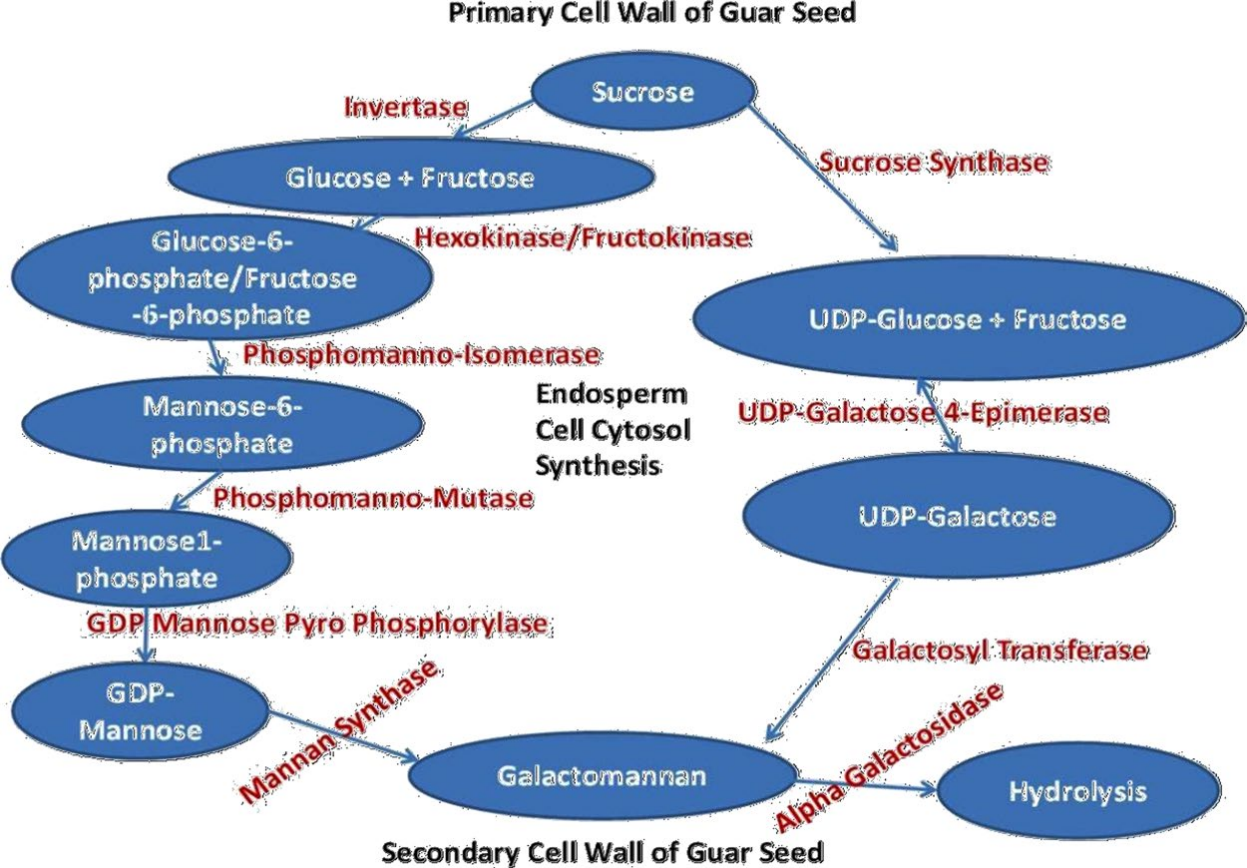
Proposed outline of galactomannan biosynthesis in endosperm cell cytosol of guar (*Cyamopsis tetragonoloba* L).

Galactomannan is the major storage polysaccharide in guar seeds and accumulates in cell walls of the endosperm, accounting for up to 26–35% of the seed dry weight. Proposed outline of galactomannan biosynthesis in endosperm cell cytosol of guar highlighting the importance of sucrose as a building block is shown in Fig. 10. In most plant species carbon is transported as sucrose. Cleavage of the *O*-glycosidic bond between the glucose and fructose units of sucrose is catalyzed by Invertase (EC 3.2.1.26) and Sucrose Synthase (EC 2.4.1.13). Invertase is a hydrolase, cleaving sucrose irreversibly into glucose and fructose, whereas Sucrose Synthase is a glycosyl transferase, converting sucrose in the presence of UDP into UDP-glucose and fructose. During seed development, entry of carbon from the maternal coat cells into the seed apoplasm is mediated by membrane-localized sugar transporters. Plant Hexokinase (EC 2.7.1.1) and Fructokinase (EC 2.7.1.4) have been shown to be involved in sugar sensing and signaling, and is proposed to be having a dual-function enzyme with both catalytic and regulatory functions. Phosphomanno-Isomerase (EC 5.3.1.8) converts fructose-6-phosphate (Fru-6-P) to mannose-6-phosphate (Man-6-P). This enzyme also functions in the reverse direction in the utilization of mannose released by hydrolysis of galactomannan on germination, after it is phosphorylated to Man-6-P. The direct precursors for galactomannan biosynthesis, GDP-D-mannose and UDP-D-galactose, are formed by the actions of GDP mannose Pyro Phosphorylase (EC 2.7.7.22) and UDP-galactose 4-epimerase (EC 5.1.3.2). Two tightly membrane-bound glycosyl transferases (Mannan synthase/Mannosyl transferase and Galactosyl transferase) together catalyze the formation of galactomannan. GDP-mannose-dependent Mannosyl Transferase (EC 2.4.1.13) transfers mannose residues to the end of the growing linear (1 → 4) β-linked mannose backbone of the galactomannan polymer. Simultaneously, UDP-galactose-dependent Galactosyl transferase (EC 2.4.1.22) transfers a galactose residue through a (1 → 6) α-linkage to a mannose at or near the non-reducing end of the growing mannan chain. Importantly, galactose cannot be transferred to preformed mannose chains. The activities of the two transferases increase in parallel during the period of galactomannan synthesis, such that the M:G ratio in the polymer remains constant. Three enzymes are involved in the hydrolysis of galactomannan during seed germination: β-mannosidase, which hydrolyses the oligomannans released by prior endo β-mannanase activity; β-mannanase, which cleaves the mannan backbone; and α-galactosidase (EC 3.2.1.22) which concomitantly removes the galactose side-chain unit.The proposed galactomannan biosynthesis pathway in the present investigation is in agreement to the pathway proposed in *Trigonella foenum graceum*^24^ and in guar variety RGC-936 seed^28^ miRNA transcriptome.

## Conclusions

The endosperm of Cluster bean, *Cyamopsis tetragonoloba* (L) seeds predominantly contains a polysaccharide, galactomannan or guar gum which has tremendous industrial applications. The mRNA was isolated from seeds collected at different developmental stages (days after anthesis), young pods, mature pods and young leaf material from two commercially important Indian guar varieties, namely, HG365 and HG870 and subjected to Illumina HiSeq. 4000 NGS Platform sequencing. Species distribution analysis showed that guar unigenes have a very high homology to several plant species and have a top blast hits with *Glycine max*, *Glycine soja*, *Medicago truncatula* and *Vigna radiata*. Annotation of assemblies with GO mapping revealed three levels of distribution, namely, Biological Processes (Metabolic; Cellular; Single organism; Biological regulation), Molecular Functions (Catalytic; Binding; Transporter) and Cellular Components (Cell; Membrane; Organelle) with respective top GO terms. The annotated contigs could be classified into five Enzyme codes; Oxidoreductases, Transferases, Hydrolases comprising 89.8%, whereas, Isomerases and Ligases comprised of 10.2%. GO studies identified major genes involved in galactomannan biosynthesis: Cellulose synthase D1 (CS) and GAUT-like gene families. Among the polysaccharide biosynthetic process (GO:0000271) genes the transcript abundance for CS was found to be predominantly more in leaf samples, whereas, the transcript abundance for GAUT-like steadily increased from 65% to 90% and above from stage1 to stage5 indicating accumulation of galactomannan in developing seeds. Similarly, relative expression pattern of two important enymes, namely, Mannan synthase and Galactosyl transferase based on RPKM values was also found to be developmentaly regulated from stage1 to stage5 indicating accumulation of galactomannan in developing seeds. The same was validated in qRT-PCR analysis where several enzymes: Invertase, Hexokinase, Phosphomannomutase, Phosphomannoisomerase, Sucrose synthase and Mannan synthase were found to exhibit low fold change gene expression in early stages (1,2) of guar seed development which gradually increased from stage3 to stage5 and in many cases was found to be highly expressed in stage8 (mature pod) convincingly proving that these enzymes were directly involved in galactomannan biosynthesis in both the varieties.The expression profile of all these enzymes were found to be higher across all stages of development in HG870. The HPLC analysis of alcohol insoluble residue extracts in both the varieties HG365 (12.98–20.66%) and HG870 (7.035–41.2%) gradually increased from stage1 stage5 (10–50 DAA) and highest accumulation occurred in mature and dry seeds with 3.8 to 7.1 fold increase, respectively. Outline of the galactomannan biosynthesis pathway has been proposed in the endopsperm cell cytosol of guar highlighting the importance of sucrose as a building block. This is the first report of transcriptome sequencing and complete profiling of guar seeds at different developmental stages (days after anthesis), young pods, mature pods and young leaf material from two commercially important Indian varieties and elucidation of galactomannan biosynthesis pathway. It is envisaged that the data presented herein will be very useful for improvement of guar through biotechnological interventions in future.

## Methods

### Collection of plant material and isolation of RNA

**Figure 11:**
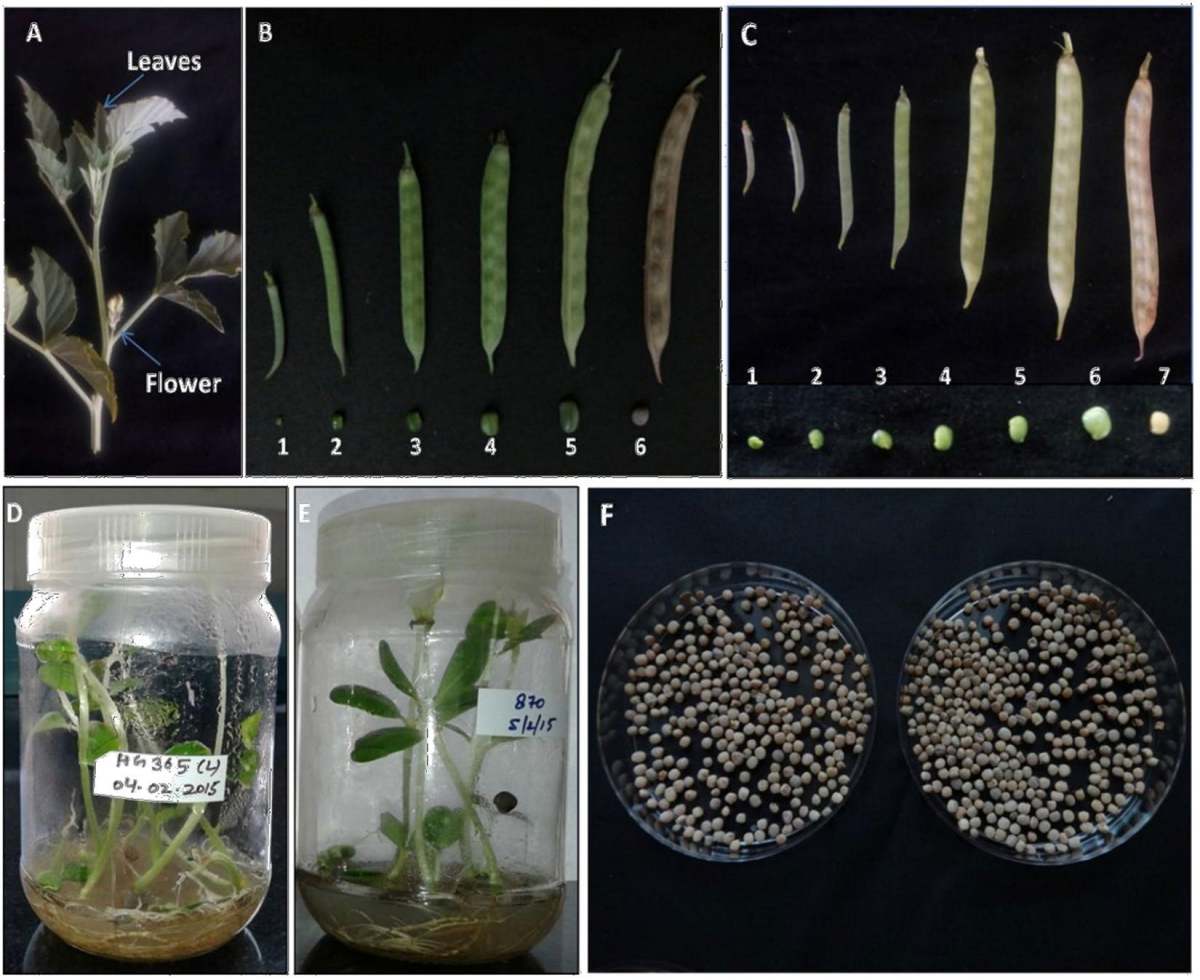
Collection of plant material from two elite commercially important Indian guar (*Cyamopsis tetragonoloba* L) varieties, namely, HG365 and HG870 sown in field employed for isolation of RNA. (**A**) Stem showing characteristic serrated leaves and pink/violet flower initiation. (**B**) Pods were collected from guar variety HG365 at different developmental stages; Days After Anthesis (DAA) e.g. 10, 20, 30, 40 and 50; and pods mature, represented by numerals 1–6, lower panel shows the seed size in respective pods. (**C**) Pods were collected from guar variety HG870 at different developmental stages; Days After Anthesis (DAA) e.g. Pods immature, 10, 20, 30, 40 and 50; and pods mature, represented by numerals 1–7, lower panel shows the seed size in respective pods. (**D**,**E**) Surface sterilized seeds of guar varieties HG365 and HG870 germinated on MS basal medium in Jam bottles in Plant Tissue Culture Laboratory maintained at 25 + 2 °C under light conditions for collection of young leaf material from 7–10-day-old germinated seedlings. (**F**) Mature seeds of guar varieties HG365 and HG870 harvested from the fields.

Two elite commercially important Indian guar (*Cyamopsis tetragonoloba* L) varieties, namely, HG365 and HG870 were sown in field in Kharif season of July-August, 2015 in fertile and sandy loam soil as shown in Supplementary Figure 9. Guar variety HG365 is early maturing (85–90 days), branched, moderate accumulator of galactomannan, whereas, the variety HG870 is early maturing (85–95 days), moderately resistant to Alternaria leaf blight, Bacterial leaf blight and root rot, high yielding and a high accumulator of galactomannan. Irrigation and minimum fertilizer application was provided as per recommended agricultural practices and harvested in October-November. Pods were collected at different developmental stages Days After Anthesis (DAA) e.g. 10, 20, 30, 40 and 50; pods young and pods mature were also collected as shown in Fig. 11. Simultaneously, surface sterilized seeds of both the varieties were germinated on MS basal medium in Jam bottles in Plant Tissue Culture Laboratory maintained at 25 + 2 °C under light conditions for collection of young leaf material from 7–10 day-old germinated seedlings as shown in Fig. 11. The collected plant material was quickly frozen in liquid nitrogen and stored at −80 °C until further use. Total RNA was isolated using RNA Sure Plant Kit Nucleo-Pore (Genetix Biotech Asia Pvt. Ltd., New Delhi, India) from elite Indian guar (*Cyamopsis tetragonoloba* L) varieties at different developmental stages of pod/seed formation (DAA) Days After Anthesis e.g. 10, 20, 30, 40 and 50, pods young, pods mature and young leaves by employing 200 mg of tissue grinded in liquid nitrogen using pestle mortar as per manufacturer’s protocol with minor modifications. Briefly, added 1.0 ml of RLB-P lysis buffer, vortex and loaded on to RNASure Shredder, centrifuged at 10,000 rpm, at 4 °C for 3 min using Sigma 4K-15, GmbH, Germany and transferred the filtrate to a new eppendorf tube. Added 700 µl of 70% ethanol, mix by pulse vortex. Take a RNASure Plant column along with collection tube and loaded the lysate on to this column for binding RNA, centrifuged at 10,000 rpm, at 4 °C for 1 min. Added 350 µl of RDB buffer for desalting and centrifuged at 10,000 rpm, at 4 °C for 1 min. Separately, added 10 µl of DNase to 90 µl of DNase reaction buffer in 1.5 ml tube for removing traces of genomic DNA, mix and apply 95 µl of it directly to silica membrane of RNASure Plant Column. Incubated at room temperature for 15 min. Added 200 µl washing buffer RWB2 buffer and centrifuged at 10,000 rpm, at 4 °C for 1 min. Discard flow through and place the RNASure Plant column to a new collection tube. Repeated the above washing step twice with first 600 µl and then with 200 µl of washing buffer RWB3 and centrifuged at 10,000 rpm, at 4 °C for 2 min discard the flow through and transferred the column to a new collection tube. Allowed the column to dry at room temperature for 3–4 min and then eluted the RNA with 50 µl of RNase free water. The mRNA was purified from total RNA using oligo-dT columns. The mRNA samples were quantified and qualified on the Agilent Technologies 2100 Bioanalyzer (Santa Clara, California, USA).

### Library preparation and transcriptome sequencing

Synthesis of double stranded cDNA and preparation of cDNA library of elite Indian Guar (*Cyamopsis tetragonoloba* L) varieties at different developmental stages of pod/seed Formation (DAA) Days After Anthesis was accomplished as per the Illumina protocol. The Transcriptome sequencing was accomplished on the Illumina HiSeq4000 NGS platform (San Diego, California, USA). Gene Expression Analysis (DEG) and Enzyme Discovery were completed with the Qiagen CLC Genomics Work Bench 9.5 (Waltham, Massachusetts, USA) – hereafter referred to as CLC. Pipelined steps are given as follows:- Raw read (paired end −100bp) files were imported in CLC WB using NCBI/Sanger illumina pipeline 1.8 or later option, reads were base quality trimmed, merged and *de novo* assembled. Assembled contigs were annotated using Blast2GO software from BioBam Bioinformatics (Valencia, Spain). Various stages of seed development of cluster bean varieties HG365 and HG870 were mapped with at least a 12 times coverage to the overall *Cyamopsis tetragonoloba* assembly with associated RPKM values. Pods young and Pods mature Transcriptome sequence were mapped to the overall *Cyamopsis tetragonoloba* assembly with associated RPKM values. Reads Per Kilobase per million Reads (RPKM) were calculated according to Mortazavi *et al*.^29^.

### RNA-Seq data analysis and *de novo* transcriptome assembly

The sequencing raw reads received in FastQ format, were processed for per base quality check by using FastQC version 0.11.4^30^. The adaptor sequences and reads having PHRED quality score less than 30 were filtered out and were not considered for transcriptome assembly. Further quality filtering and trimming of the raw reads was obtained by Trimmomatic software version 0.36^31^.The clean reads were imported to Qiagen CLC Genomics Work Bench 9.5 (Waltham, Massachusetts, USA) using NCBI/Sanger illumina pipeline 1.8 or later option, and were then *de novo* assembled with parameters given in Supplementary Table 1. Further processing was done by cleaning the transcripts by using perl script SeqClean (https://sourceforge.net/projects/seqclean/) in order to remove rRNA sequences, low-complexity RNA and polyA stretches. Subsequently, CD-HIT software version 4.6.1^32^ was employed to cluster the transcripts and thereby remove redundancies and obtain non-redundant unigenes. For which the sequence identity parameter was set at 0.95 (Altschul *et al*., 1990). The estimation for the expression level of unigenes (Pods young and Pods mature), FPKM (Fragments per kilobase of transcript per millions fragments sequenced) was performed by RSEM (RNA-Seq by Expectation Maximization). The unigenes having FPKM values less than 1 and length less than 200 bp were removed, to assure that the resulting assembly was of good quality^33^.

### Functional annotation of cyamopsis tetragonoloba pods transcriptome

The resulting unigenes were BLAST searched against the public database. The BLASTX tool^34^ was employed to carry out the sequence similarity search of unigenes against NCBI non-redundant protein (Nr) database with the help of Blast2GO tool version 3.1.3^35^ and a cut-off *E*-value set at 1e^−5^. Blast2GO suite was employed to carry out InterproScan (IPS) Search, to map and fetch the Gene ontology terms (GO) and unique enzyme code (EC) annotations of unigenes from seeds collected at different developmental stages, pods young, pods mature and young leaf samples of *Cyamopsis tetragonoloba*. Later the WEGO tool^36^ was employed to carry out the functional classification of the genes present in the transcriptome into different categories like Biological Processes, Molecular Functions and Cellular Components. Blast2GO tool was also used for the annotation of unigenes against Kyoto Encyclopedia of Genes and Genomes (KEGG) metabolic pathways database^37^.

### Differential gene expression analysis

The Bioconductor package edgeR (Extraction of Differential Gene Expression R Package 3.3.1) was used to extract differentially expressed genes (DEGs) between different stages of seeds/pod development. The *p*-value of ≤0.05 and two-fold change (*C* value ≥ 1) was set as the threshold for the identification of DEGs.

### Functional classification and biological pathway elucidation

#### Designing of PCR primers

Twenty two sets of forward and reverse primers of Gene of Interest were designed for selective members of gene families of enzymes potentially involved in galactomannan biosynthesis pathway and three sets Housekeeping gene primers, namely SPT5, HPATcp and PAGM from the transcriptiome data were designed as shown in Table 1 and Supplementary Table 2.

### Synthesis of cDNA and validation of PCR primers

Two elite Indian guar (*Cyamopsis tetragonoloba* L) varieties, namely, HG365 and HG870 were sown in field in Kharif season of July-August, 2016 as described earlier. Total RNA was isolated using RNA Sure Plant Kit Nucleo-Pore (Genetix Biotech Asia Pvt. Ltd., New Delhi, India) from elite Indian guar (*Cyamopsis tetragonoloba* L) varieties at different developmental stages of pod/seed formation (DAA) Days After Anthesis e.g. 10, 20, 30, 40 and 50, pods young, pods mature and young leaves by employing 200 mg of tissue as described earlier. A 2 µg of template RNA was used for the synthesis of cDNA using QuantiTect Reverse Transcription kit (Qiagen, Hilden, Germany) in a two step method as per manufacturer’s protocol in a total reaction volume of 40 µl. Briefly, added 4 µl of gDNA wipe-out buffer 7X, 2 µg of template RNA, and the volume was made to 28 μl with RNase free water followed by incubation at 42 °C for 2 min and immediately placed on ice. Prepared Reverse transcription master mix on ice by taking 2 µl of Reverse transcription master mix (Quantiscript Reverse Transcriptase), 8 µl of Quantiscript RT buffer 5X, 2 µl RT primer mix. Added 28 μl of the reaction mix from step one to the above tube containing 12 µl of Reverse transcription master mix. Mixed and incubated at 42 °C for 15 min, followed by incubation at 95 °C for 3 min. The cDNA synthesized was qualified and quantified using agarose gel electrophoresis and NanoDrop spectrophotometer ND1000 (Thermo Scientific, Wilmington, Delaware) and stored at −20 °C until further use.

An aliquot of 0.5 µl of cDNA was used as a template for PCR amplification using twenty two different set of primers designed for selective members of gene families potentially involved in galactomannan biosynthesis pathway described above. Amplifications were carried out in peqStar 2X gradient Thermocycler (VWR International GmbH, Germany) programmed for initial denaturation at 94 °C for 2 min, denaturation at 94 °C for 45 sec, followed by 36 cycles each consisting of initial denaturation at 94 °C for 2 min, annealing at 52–54 °C for 1 min and elongation at 72 °C for 2 min. Final extension step included 72 °C incubation for 10 min, and soak at 4 °C.

PCR reaction was carried out in 20 μl of reaction mixture, containing 10 ng of template cDNA, 0.2 mM of each dNTP (Promega Corporation, WI, USA), 1X GoTaq flexi buffer (Promega Corporation, WI, USA), 1 mM MgCl_2_ solution (Promega Corporation, WI, USA), 0.25 μM of each primer (Eurofins Genomics India Pvt. Ltd., Bengaluru, India) and 1.25U GoTaq DNA polymerase (Promega Corporation, WI, USA), along with appropriate volume of nuclease free water. The amplified products were resolved (Bio-Rad Research Laboratories, USA) on ethidium bromide stained agarose gels (2.5%) in 1X TAE buffer at 50 V along with 100 bp ladders. The gels were visualized on trans-UV illuminator and photographed in gel documentation system (Syngene, UK).

### qRT-PCR analysis

For quantitative real time PCR analysis QuantiTect SYBR Green PCR kit (Qiagen, Hilden, Germany) was employed using Rotor GeneQ. 5plex HRM cycler. The machine was programmed for initial denaturation at 95 °C for 15 min, denaturation at 94 °C for 15 sec, followed by 36 cycles each consisting of initial denaturation at 94 °C for 15 sec, annealing at 52–54 °C for 30 sec and elongation at 72 °C for 30 sec. The qPCR reaction mixture contained 10 ng of template cDNA, 1X QuantiTect SYBR Green PCR master mix (Qiagen, Hilden, Germany), 0.25 μM of each primer (Eurofins Genomics India Pvt. Ltd., Bengaluru, India) and the volume was made to 20 μl with RNase free water in 0.1 ml Strip tubes with caps (Qiagen, Hilden, Germany). Twenty two different set of primers designed for selective members of gene families potentially involved in galactomannan biosynthesis pathway were employed. The qRT-PCR batch experiments were performed by employing one set of primers designed for each of the selective members of gene families of enzyme across different developmental stages of pod/seed formation (DAA) Days After Anthesis e.g. 10, 20, 30, 40 and 50, leaves, pods young and pods mature coded as stage 1, 2, 3, 4, 5, 6, 7 & 8 from two elite Indian guar (*Cyamopsis tetragonoloba* L) varieties, namely, HG365 and HG870. Three housekeeping genes were designed from Transcriptome sequence contigs data and employed as housekeeping or reference genes and got synthesized from Sigma-Aldrich, India (Merck). These were transcription elongation factor SPT5 (Ct_1828), histidinol-phosphate chloroplastic-like gene HPATcp (Ct_2645) and phospho acetyl glucosamine mutase gene PAGM (Ct_14209). The housekeeping gene which gave fairly low and stable average CT values across different developmental stages of pod/seed formation from stage 1 to 8 was employed for gene expression analysis. On the other hand, commonly used housekeeping genes such as Actin, Ubiquitin or β-globin were also designed from the Transcriptome data and employed for qRT-PCR analysis. However, these three HK genes gave fairly high CT values along with lot of inconsistency so were not employed in present investigation (data not shown). Melt curve analysis was performed from 65 °C to 95 °C for verification of amplicon specificity. The experiment was repeated with two biological replicates and the average CT values of Housekeeping gene HK were deducted from average CT values of Gene of Interest for twelve enzymes with primer code 1 to 21 involved in Galactomannan biosynthesis giving ΔCT. The values so obtained were employed for subtracting the CT values of calibrator which was taken as stage 7 young pods for obtaining ΔΔCT. Thus, CT values of Gene of interest were first normalized by phospho acetyl glucosamine mutase gene (PAGM) served as a Housekeeping gene and further normalized from stage 7 young pod values by employing the 2^ΔΔCT^ method of Livak and Schmittgen^38^ for calculating fold change expression.

### Quantification of galactomannan

Estimation of galactomannan from two elite Indian Guar (*Cyamopsis tetragonoloba* L) varieties, namely, HG365 and HG870 at different developmental stages of pod/seed formation (DAA) Days After Anthesis e.g. 10, 20, 30, 40, 50 and dry seed was accomplished using HPLC RI detector from Oniosome Health Care Pvt. Ltd., Mohali, Punjab, India by following the protocol given for *Trigonella foenum graceum*^24^ with minor modifications. Seeds samples at different developmental stages, namely, Stage 1, 2, 3, 4, 5 and dry seeds and endosperm of stage 2, 3, 4, 5, dry seeds and seed coat were employed for quantification of galactomannan in Alcohol Insoluble Residues (AIR) as shown in Supplementary Figure 10. Briefly, five seeds/endosperm/seed coat at different developmental stages were randomly collected, grinded in liquid nitrogen using pestle mortar and transferred the powder to 2.0 ml screw cap tubes. Added 1.8 ml 70% ethanol, mixed, briefly vortexed and incubated at 65 °C for one h followed by centrifugation at 10,000 rpm for 5 min. Discard supernatant and resuspended pellet in 1.0 ml 70% ethanol, mixed followed by centrifugation at 10,000 rpm for 5 min. This step was repeated twice and the AIR pellet so derived was subjected to lyophilization by vaccum drying over night. The alcohol insoluble residue pellet was weighed and resuspended in 1.0 ml HPLC grade water for further analysis. The samples were diluted and filtered through 0.22 µM Whatman filter membrane. Analysis of sugars was performed by injecting 20 µl of sample and analyzed using HPLC Agilent 1200 series equipped with Refractive Index Detector having Amino bonded column (250 mm × 4.5 mm, 5 µM) with flow rate of 1.0 ml min^−1^ Column temperature of 65 °C using mobile phase of Acetonitrile: water (80:20 v/v). The standards for galactomannan and other sugars were purchased from Sigma-Aldrich, India. The chromatograms obtained were used first for estimation of amount of galactomannan in each of the sample by taking into account sample area, dilution and slope of curve; and percentage of galactomannan was calculated by dividing the amount of galactomannan obtained in each sample by the initial sample weight. The experiment was repeated twice and the average values were plotted for percentage galactomannan at different stages of seed development.

## Supplementary information

Supplementary Figs and Tables

## Data Availability

SRA Bio-Project: SRA submission ID: SUB1615029; SRA Accession: SRP077589 “*Cyamopsis tetragonoloba*: Seed Development Expression of RNA and Genome sequencing” June 17, 2016 in NCBI, USA at http://www.ncbi.nlm.nih.gov/sra

## References

[1] Hymowitz T, Matlock RS (1963). Guar in the United States. Oklahoma Agri Expt. Stat. Bull. B.

[2] Dhugga KS (2004). Guar seed β-mannan synthase is a member of the cellulose synthase super gene family. Science.

[3] Naoumkina M (2007). Analysis of cDNA libraries from developing seeds of guar *(Cyamopsis tetragonoloba* (L.) Taub). BMC Plant Biol..

[4] Naoumkina M (2008). Metabolic and genetic perturbations accompany the modification of galactomannan in seeds of Medicago truncatula expressing mannan synthase from guar (*Cyamopsis tetragonoloba* L.). Plant Biotech. J..

[5] Ng D (2009). An improved technique for concentration measurement of galactomannan solutions by differential refractive index. Carbohyd. Poly..

[6] Brahmi P, Bhat KV, Bhatnagar AK (2009). Study of allozyme diversity in guar [*Cyamopsis tetragonoloba* (L.) Taub.] germplasm. Genet. Res. Crop Evol..

[7] Punia A, Arora P, Yadav R, Chaudhury A (2009). Optimization and inference of PCR conditions for genetic variability studies of commercially important cluster bean varieties by RAPD analysis. Asia Pac J. Mol. Biol. Biotechnol..

[8] Punia A, Yadav R, Arora P, Chaudhury A (2009). Molecular and morphological characterization of superior cluster bean (*Cyamopsis tetragonoloba*) Varieties. J. Crop Sci.Biotechnol..

[9] Kuravadi NA, Tiwari PB, Choudhary M, Randhawa GS (2013). Genetic diversity study of cluster bean (*Cyamopsis tetragonoloba* (L.) Taub) landraces using RAPD and ISSR markers. Int J. Adv. Biotechnol. Res..

[10] Sharma P, Kumar V, Raman KV, Tiwari K (2014). A set of SCAR markers in cluster bean (*Cyamopsis tetragonoloba* L.Taub) genotypes. Adv. Biosci Biotechnol..

[11] Kumar S (2016). Development and validation of EST-derived SSR markers and diversity analysis in cluster bean (*Cyamopsis tetragonoloba*). J. Plant Biochem. Biotechnol..

[12] Yamada K (2003). Empirical analysis of transcriptional activity in the. Arabidopsis genome Science.

[13] Bertone P (2004). Global identification of human transcribed sequences with genome tiling arrays. Science.

[14] David L (2006). A high-resolution map of transcription in the yeast genome. Proc. Natl. Acad. Sci. USA.

[15] Schuster SC (2008). Next–generation sequencing transforms today’s biology. Nat. Methods..

[16] Wang Z, Gerstein M, Snyder M (2009). RNA-Seq: a revolutionary tool for transcriptomics. Nat. Rev. Genet..

[17] Zenoni S (2010). Characterization of transcriptional complexity during berry development in Vitis vinifera using RNA-Seq. Plant Physiol..

[18] Cheung F (2006). Sequencing Medicago truncatula expressed sequenced tags using 454 Life Sciences Technology. BMC Genomics.

[19] Wang Y (2012). Exploring the switchgrass transcriptome using second generation sequencing technology. PLoS One.

[20] Meyer E, Logan TL, Juenger TE (2012). Transcriptome analysis and gene expression atlas for *Panicum hallii* var. filipes, a diploid model for biofuel research. Plant J.

[21] Xie F (2012). De novo sequencing and a comprehensive analysis of purple sweet potato (*Impomoea batatas* L.) transcriptome. Planta.

[22] Wang Z (2011). Characterization and development of EST-derived SSR markers in cultivated sweetpotato (*Ipomoea batatas*). BMC Plant Biol..

[23] Wang Z (2014). Development and characterization of simple sequence repeat (SSR) markers based on RNA-sequencing of *Medicago sativa* and in silico mapping onto the. M. truncatula genome. PLoS ONE.

[24] Wang Y, Alonso AP, Wilkerson CG, Keegstra K (2012). Deep EST profiling of developing fenugreek endosperm to investigate galactomannan biosynthesis and its regulation. Plant Mol. Biol..

[25] Tanwar UK, Pruthi V, Randhawa GS (2017). RNA-Seq of guar (*Cyamopsis tetragonoloba* L. Taub.) leaves: De novo transcriptome assembly, functional annotation and development of genomic resources. Front. Plant Sci..

[26] Kaila T (2017). Chloroplast genome sequence of cluster bean (*Cyamopsis tetragonoloba* L.): Genome structure and comparative analysis. Genes..

[27] Rawal HC (2017). High quality unigenes and microsatellite markers from tissue specific transcriptome and development of a database in cluster bean (*Cyamopsis tetragonoloba* (L.) Taub). Genes.

[28] Tyagi A (2018). Genome-wide discovery of tissue-specific miRNAs in cluster bean (*Cyamopsis tetragonoloba*) indicates their association with galactomannan biosynthesis. Plant Biotechnol. J..

[29] Mortazavi A, Williams BA, McCue K, Schaeffer L, Wold B (2008). Mapping and quantifying mammalian transcriptome by RNA-seq. Nat Methods.

[30] Andrews, S. FastQC: A Quality Control Tool for High Throughput Sequence Data. Available online at: http://www.bioinformatics.babraham.ac.uk/projects/fastqc/ (2010).

[31] Bolger AM, Lohse M, Usadel B (2014). Trimmomatic: A flexible trimmer for Illumina sequence data. Bioinformatics.

[32] Li W, Godzik A (2006). Cd-hit: A fast program for clustering and comparing large sets of protein or nucleotide sequences. Bioinformatics.

[33] Li B, Dewey CN (2011). RSEM: Accurate transcript quantification from RNA-Seq data with or without a reference genome. BMC Bioinfo..

[34] Altschul SF, Gish W, Miller W, Myers EW, Lipman DJ (1990). Basic local alignment search tool. J. Mol. Biol..

[35] Conesa A (2005). Blast2GO: A universal tool for annotation, visualization and analysis in functional genomics research. Bioinformatics.

[36] Ye J (2006). WEGO: a web tool for plotting GO annotations. Nucleic Acids Res..

[37] Kanehisa M, Goto S (2000). KEGG: Kyoto Encyclopedia of Genes and Genomes. Nucleic Acids Res..

[38] Livak, K. J. & Schmittgen, T. D. Analysis of relative gene expression data using real-time quantitative PCR and the 2(−Delta Delta C(T))* Method.* PMID:11846609 402–408 (2001). 10.1006/meth.2001.1262 11846609

