# Supplementary material for: Elucidation of Galactomannan Biosynthesis Pathway Genes through Transcriptome Sequencing of Seeds Collected at Different Developmental Stages of Commercially Important Indian Varieties of Cluster Bean (Cyamopsis tetragonoloba L.)
Source: Sci Rep. 2019 Aug 8;9:11539. doi: 10.1038/s41598-019-48072-w (PMC6687724; doi:10.1038/s41598-019-48072-w)
Supplement: Supplementary file 1 — Supplementary Figs and Tables [file 41598_2019_48072_MOESM1_ESM.pdf]

Elucidation of Galactomannan Biosynthesis Pathway Genes through Transcriptome Sequencing of Seeds Collected at Different Developmental Stages of Commercially Important Indian Varieties of Cluster Bean (*Cyamopsis tetragonoloba* L.)

Ashok Chaudhury[1, *] •Tanvi Kaila[2] • Kishor Gaikwad[2]
[1]Plant Molecular Biology Laboratory, Department of Bio and Nano Technology, Bio and Nano Technology Centre, Guru Jambheshwar University of Science and Technology, Hisar-125 001, Haryana, India
[2]ICAR-National Research Centre on Plant Biotechnology, LBS Centre, Pusa Campus, New Delhi-110 012, India
[*]Corresponding Author
Phone: +91-1662-263306
Fax: +91-1662-276240
E-mail: ashokchaudhury@hotmail.com

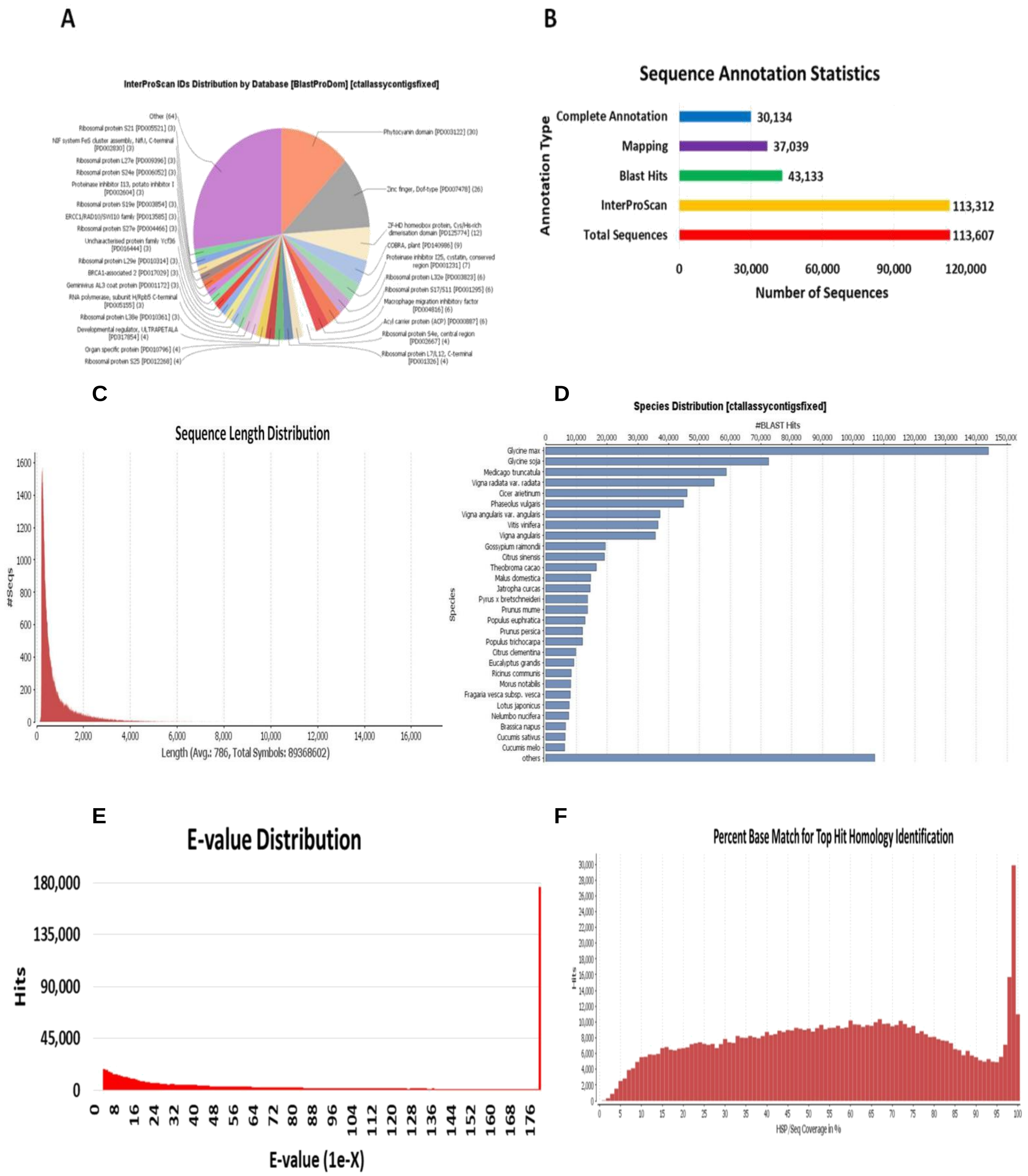

Suppl. Fig. 1. Distribution and functional annotation of unigenes of *Cyamopsis tetragonoloba*. (A) Distribution of *de novo* assembled contigs based on InterProScan IDs assigned. (B) Sequence Annotation statistics. (C) Sequence length distribution. (D) Species distribution. (E) E-value distribution. (F) Top hit percent base match percentage.

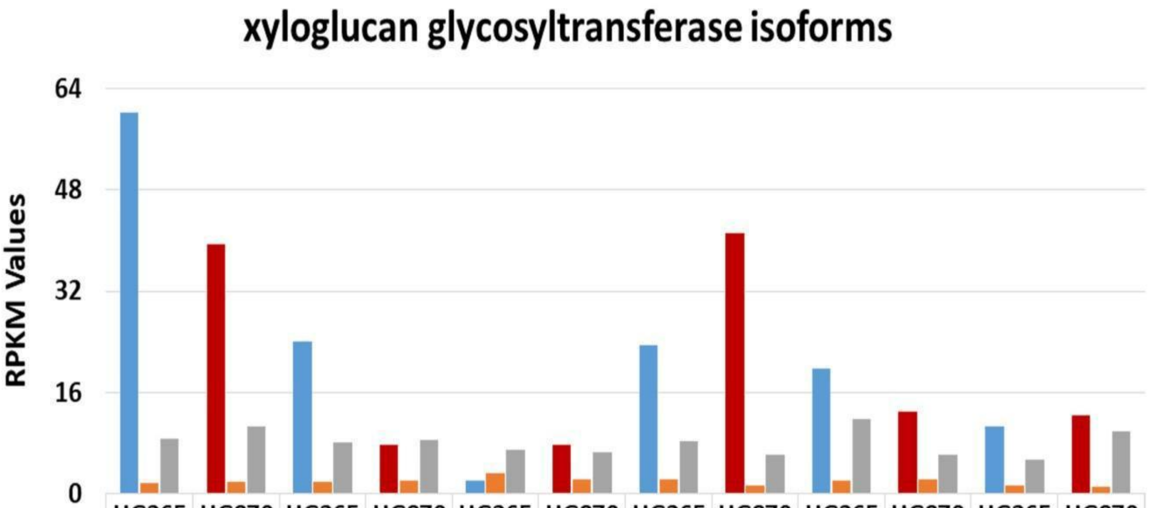

	HG365 Stage1	HG870 Stage1	HG365 Stage2	HG870 Stage2	HG365 Stage3	HG870 Stage3	HG365 Stage4	HG870 Stage4	HG365 Stage5	HG870 Stage5	HG365 Stage7	HG870 Stage7
CSLC4	60.29	39.46	24.03	7.7	2.02	7.62	23.52	41.23	19.79	12.85	10.59	12.33
CSLC5	1.76	1.84	1.91	2.15	3.16	2.23	2.18	1.26	2	2.3	1.35	1.05
CSLC5	8.69	10.69	7.99	8.48	6.88	6.53	8.21	6.05	11.69	6.17	5.29	9.87

Suppl. Fig. 2. Relative expression of identified xyloglucan glycosyltransferase isoforms of *Cyamopsis tetragonoloba* contigs assembled from all sequencing reads.

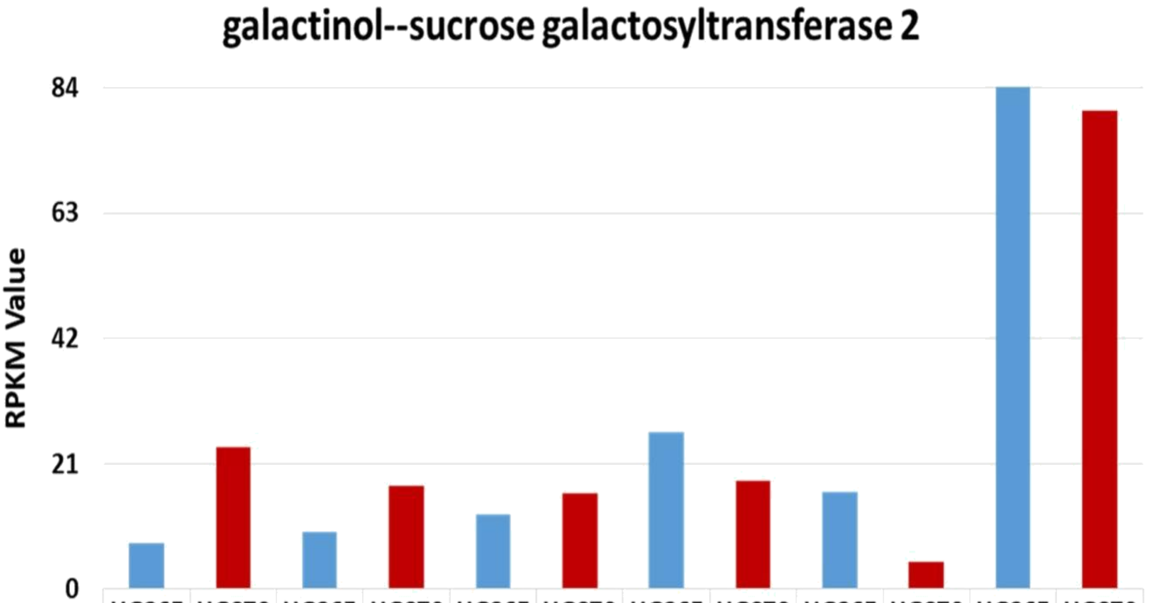

	HG365 Stage1	HG870 Stage1	HG365 Stage2	HG870 Stage2	HG365 Stage3	HG870 Stage3	HG365 Stage4	HG870 Stage4	HG365 Stage5	HG870 Stage5	HG365 Stage7	HG870 Stage7
AGA2	7.67	23.83	9.51	17.27	12.37	16.05	26.2	18.12	16.21	4.59	99.38	80.09

Suppl. Fig. 3. Relative expression of identified galactinol sucrose glycosyltransferase 2 transcripts of *Cyamopsis tetragonoloba* contigs assembled from all sequencing reads.

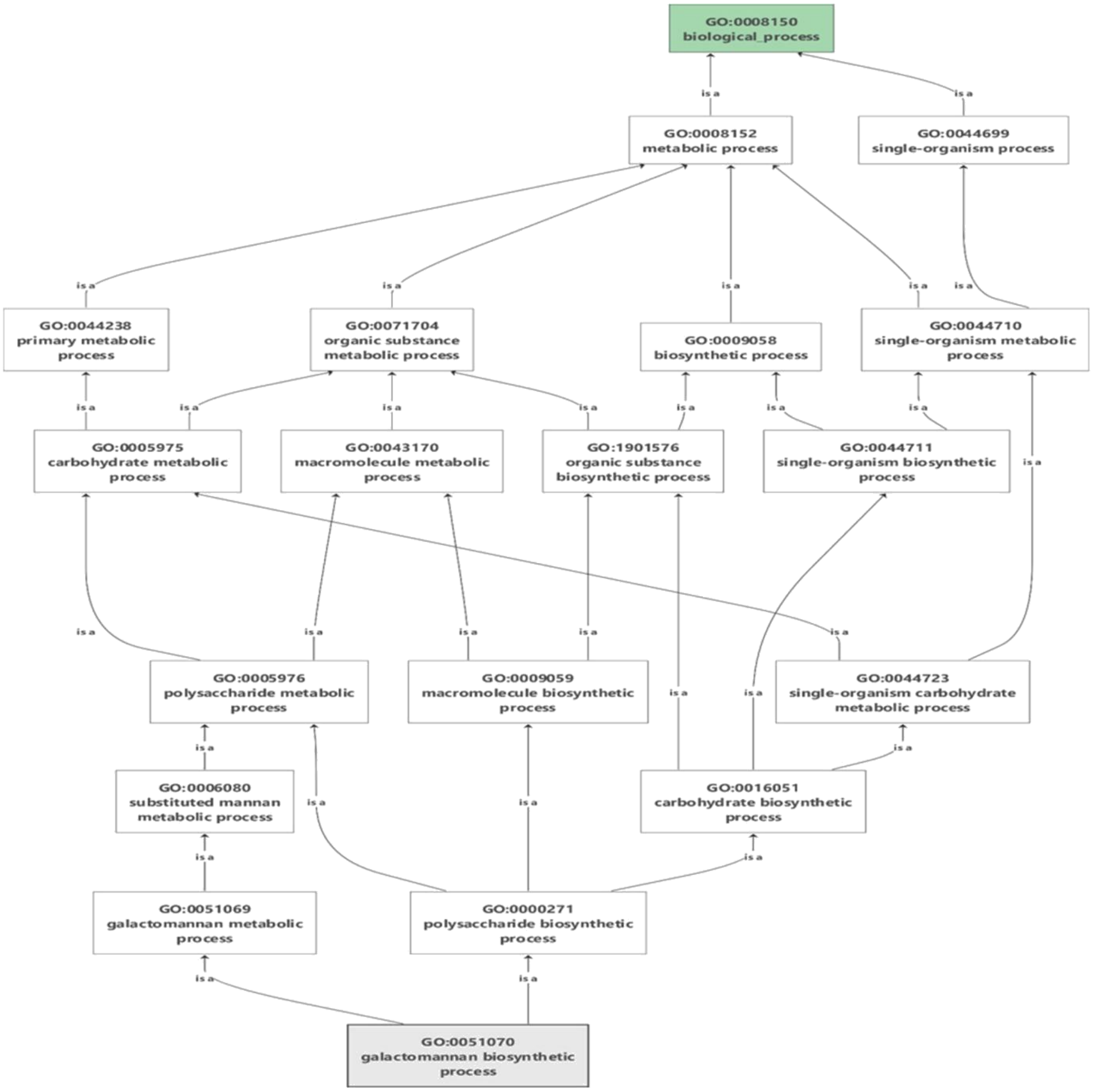

Suppl. Fig. 4. Kyoto Encyclopedia of Genes and Genomes (KEGG) pathway enrichment of DEGs.

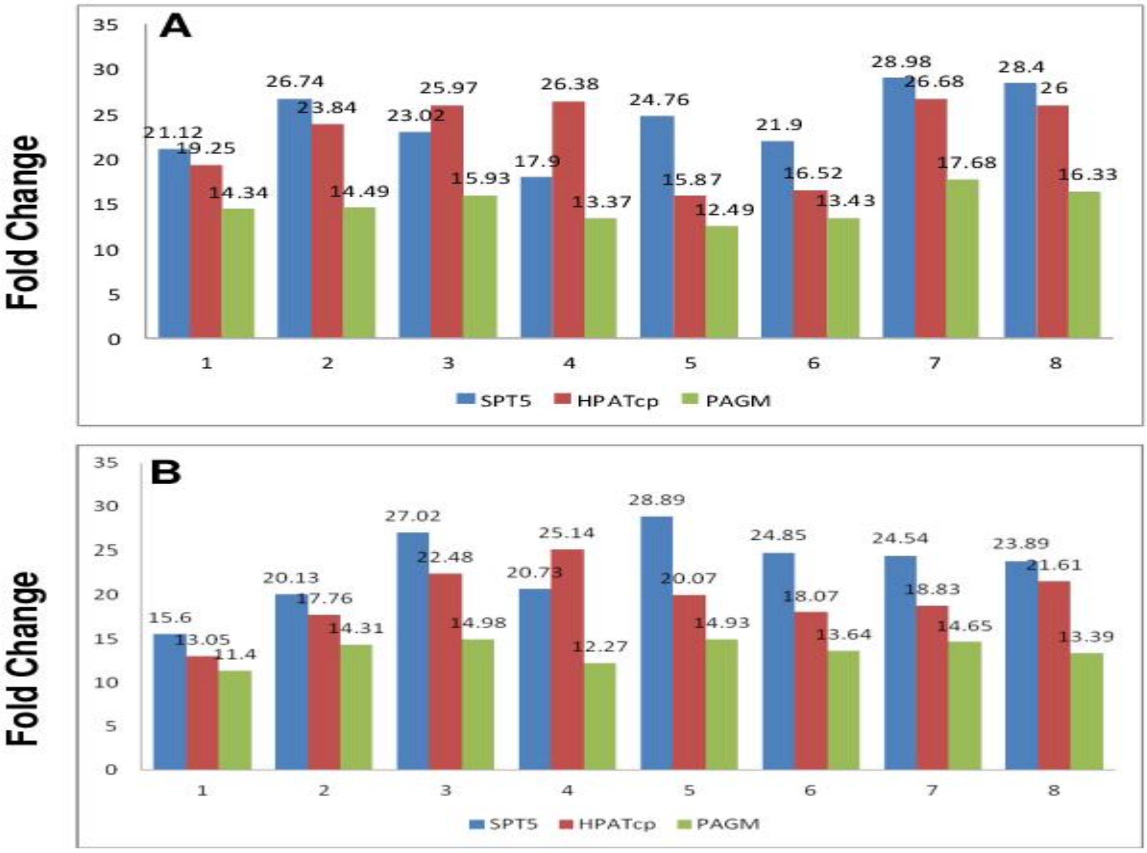

Suppl. Fig. 5. The average CT values of the three housekeeping genes, namely, SPT5, HPATcp and PAGM employed for determining expression profile of genes of interest involved in Galactomannan biosynthesis from two elite commercially important Indian Guar (*Cyamopsis tetragonoloba* L) varieties, namely, (A) HG365 and (B) HG870 at different developmental stages; Days After Anthesis (DAA) e.g. 10, 20, 30, 40 and 50 leaf, pods young, pods mature (denoted by numerals 1-8).

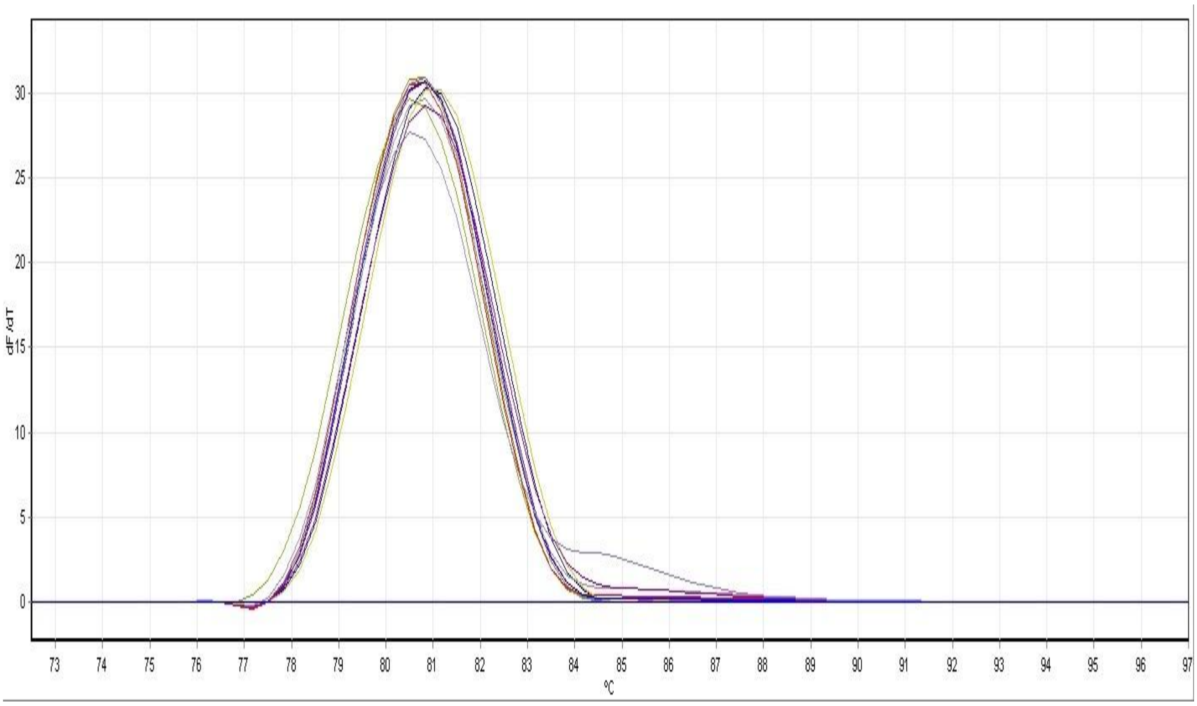

Suppl. Fig. 6. Melt curve of PAGM housekeeping gene for normalizing the data across eight developmental stages of pod/seed formation, leaf, pods young, pods mature in qRT-PCR analysis of guar varieties, namely, HG365 and HG870

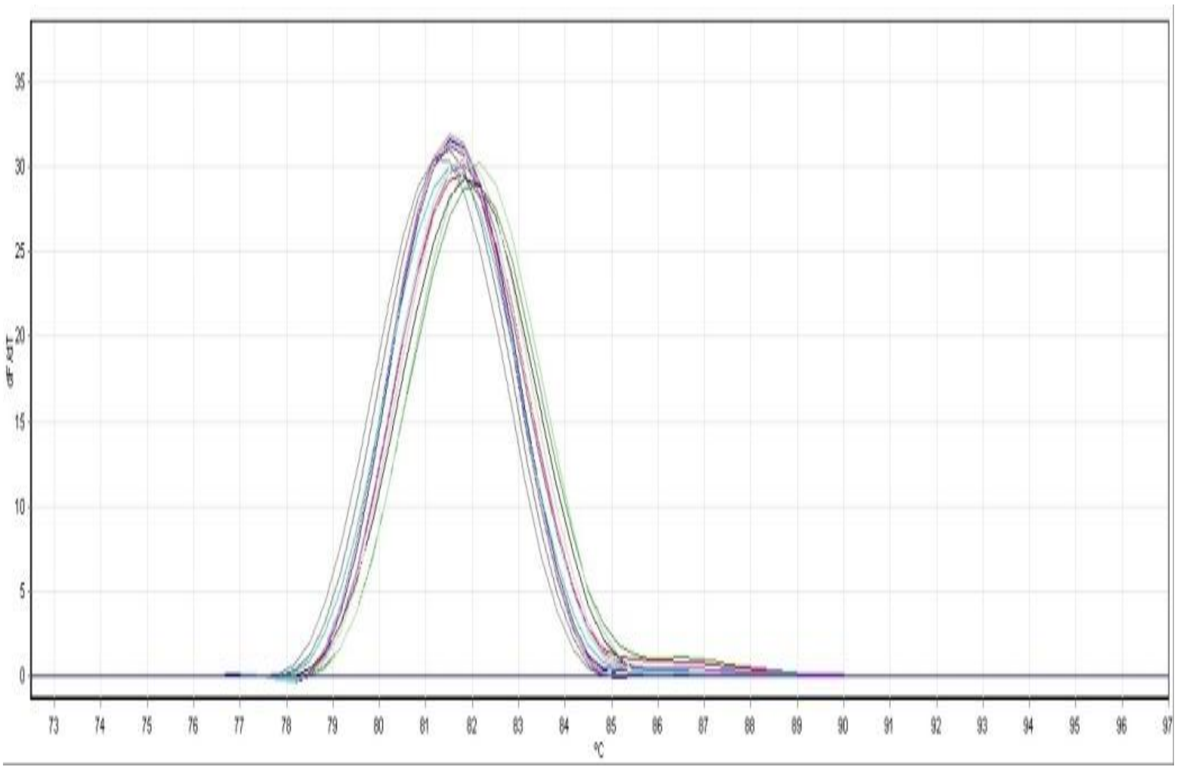

Suppl. Fig. 7. Melt curve of representative enzymes involved in galactomannan biosynthesis for gene of interest Ct_2106; Sucrose synthase, Primer Code 10 (152bp) and Ct_12647 and Mannan synthase, Primer Code 11 (144bp) across eight developmental stages of pod/seed formation, leaf, pods young, pods mature in qRT-PCR analysis of guar varieties, namely, HG365 and HG870.

A. Primer 3

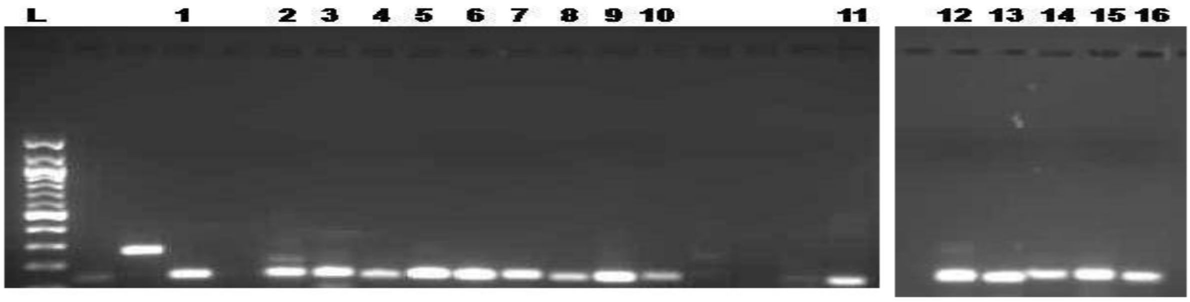

B. Primer 10

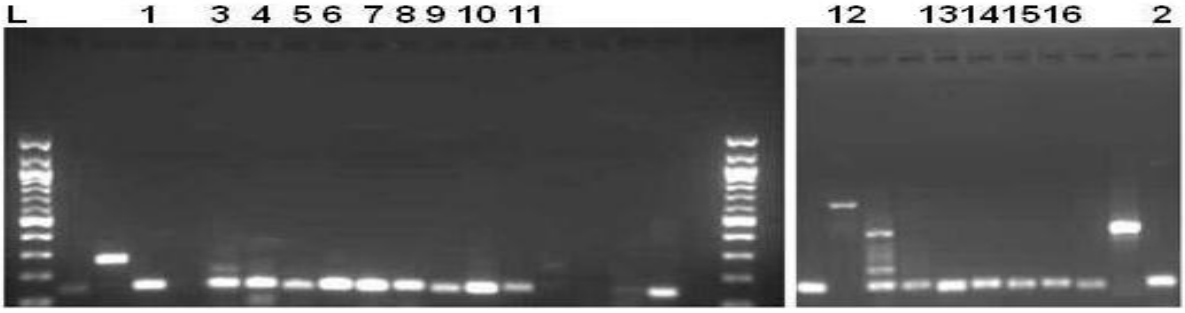

C. Primer 11

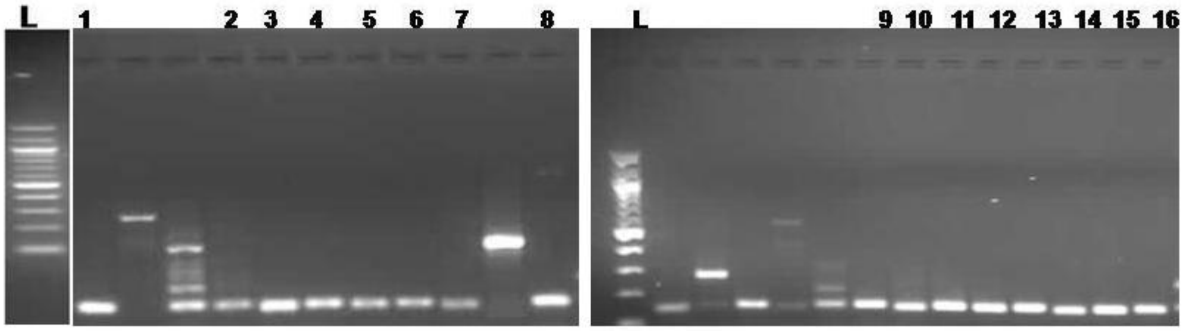

D. Primer 13

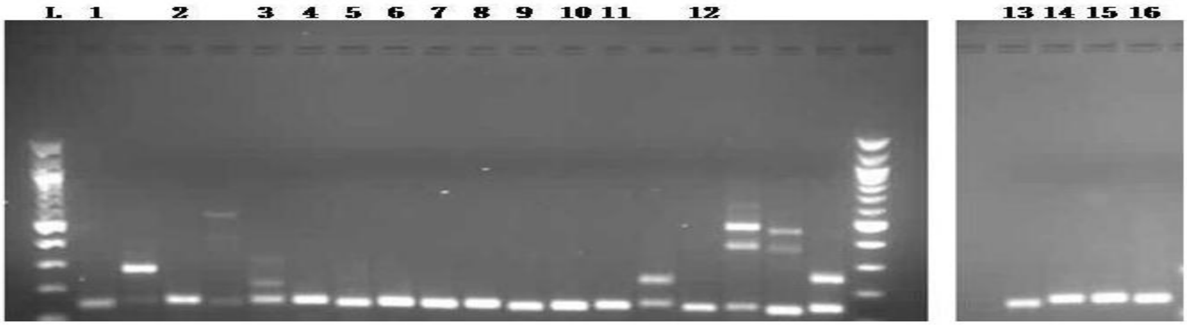

Suppl. Fig 8. Full length images of gel pictures for validation of PCR analysis of primers (Primer code 3, 10, 11 and 13) for gene families or enzymes involved in Galactomannan biosynthesis from two elite commercially important Indian Guar (*Cyamopsis tetragonoloba* L) varieties, namely, HG365 and HG870 at different developmental stages as shown in Fig. 6.

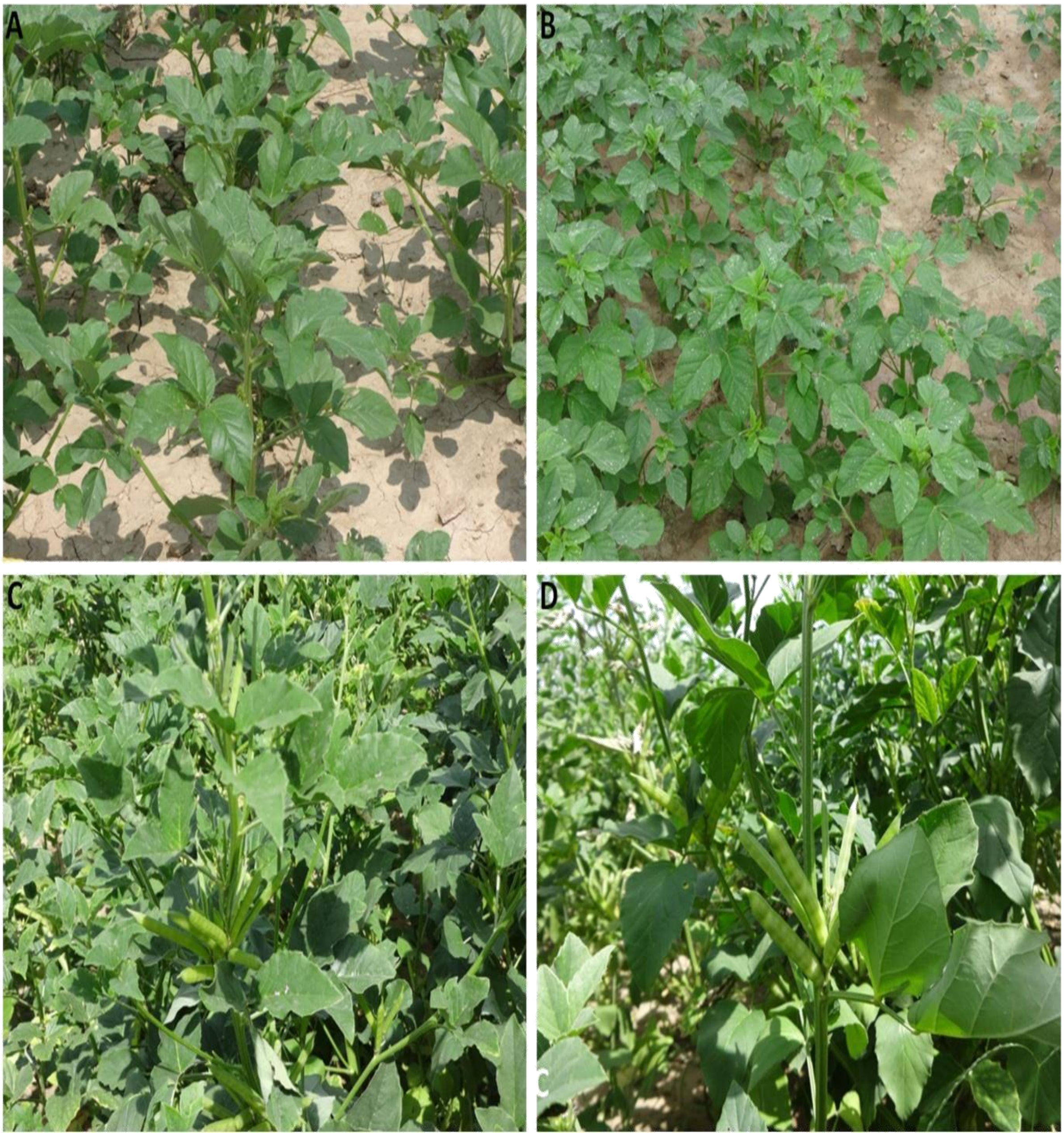

Suppl. Fig. 9. Two elite commercially important Indian guar (*Cyamopsis tetragonoloba* L) varieties, namely, HG365 and HG870 sown in field in Kharif season of July-August, 2015 & 2016 in fertile and sandy loam soil. (A) Standing crop of HG365. (B) HG870 in vegetative phase and flower induction phase. (C) Standing crop of HG365 and (D) HG870 showing pods at different developmental stages.

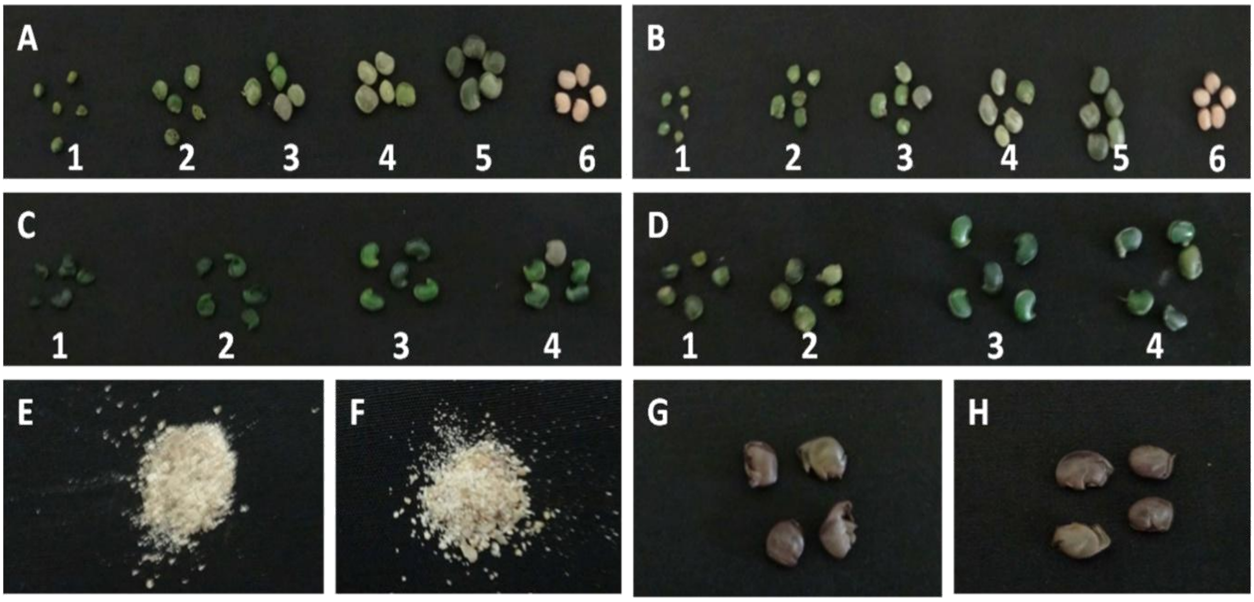

Suppl. Fig. 10. Collection of plant material from two elite commercially important Indian guar (*Cyamopsis tetragonoloba* L) varieties, namely, HG365 and HG870 sown in field employed for isolation of Alcohol Insoluble Residue (AIR) for estimation of selected galactomannan through HPLC. Seeds from pods collected from guar varieties at different developmental stages; Days After Anthesis (DAA) e.g. 10, 20, 30, 40 and 50; dry seeds; represented by numerals 1-6; (A) HG365; (B) HG870. Endosperm of seeds from pods collected from guar varieties at different developmental stages; Days After Anthesis (DAA) e.g. 20, 30, 40 and 50; represented by numerals 1-4; (C) HG365; (D) HG870 and dry seeds; (E) HG365; (F) HG870. Seed coats of 50 DAA samples; (G) HG365; (H) HG870.

Suppl. Table 1. Assembly parameters incorporating 14 individual read files representing two varieties and 7 developmental stages.

Assembly Parameters
Mapping mode = Map reads back to contigs (slow)
Update contigs = Yes
Automatic bubble size = Yes
Minimum contig length = 200
Automatic word size = Yes
Perform scaffolding = Yes
Auto-detect paired distances = Yes
Mismatch cost = 2
Insertion cost = 3
Deletion cost = 3
Length fraction = 0.8
Similarity fraction = 0.9
Create list of un-mapped reads = No
Word size: 26
Bubble size: 50

Suppl. Table 2. Real Time qRT-PCR/PCR Primers for three Housekeeping genes, namely, SPT5, HPATcp and PAGM from two elite commercially important Indian Guar (*Cyamopsis tetragonoloba* L) varieties, namely, HG365 and HG870.

1. **Transcription elongation factor SPT5 homolog 1 gene Ct_1828**

S No	Name of primer	5'-3' Sequence	T_m value (°C)
1	TEF_SPT5 Forward	TGGACAGCCTATGACACCAA	64.2
2	TEF_SPT5 Reverse	TTCGTTGTCCCCACCTAAAG	63.7

2. **Histidinol-phosphate chloroplastic-like gene Ct_2645**

S No	Name of primer	5'-3' Sequence	T_m value (°C)
1	HPATcp Forward	TTTGACGCTGCAGTTAATGG	63.7
2	HPATcp Reverse	ATGCATTTGGGCTTCTCTTG	64.0

3. **Phosphor acetyl glucosamine mutase gene Ct_14209**

S No	Name of primer	5'-3' Sequence	T_m value (°C)
1	PAGM Forward	AACCAAGCAGTTGGAGATGC	64.2
2	PAGM Reverse	GCTGCCTACTGGGTAAATCG	63.4
